# Fabrication and characterization of eco-friendly PVA/chitosan/TEA-TOS composites as a potential light-absorbing material for energy storage and optoelectronics

**DOI:** 10.1038/s41598-026-64510-y

**Published:** 2026-08-31

**Authors:** H. Elhendawi, R. M. Ramadan, A. M. Ali, T. Fahmy

**Affiliations:** 1 Basic Science Department, Faculty of Engineering, Delta University for Science and Technology, Gamasa, 35712 Egypt; 2 Microwave Physics and Dielectrics Department, Physics Research Institute, National Research Centre (NRC), Dokki, Giza, 12622 Egypt; 3 Physics Department, Faculty of Science, Mansoura University, Mansoura, 35516 Egypt; 4 Polymer Research Group, Physics Department, Faculty of Science, Mansoura University, Mansoura, 35516 Egypt

**Keywords:** PVA/Cs, Urbach energy, Band gap, Photoconductivity, Dielectric constant, Chemistry, Materials science, Nanoscience and technology, Optics and photonics, Physics

## Abstract

The need for efficient and reliable multifunctional optical and optoelectronic devices is driving the development of new materials. In this work, a novel eco-friendly biopolymer composite based on PVA, chitosan, and the ionic liquid [TEA-TOS] is developed as an efficient light-absorbing material for energy storage and optoelectronic applications. PVA/chitosan/[TEA-TOS] composites are successfully prepared using casting technique. The molecular structure and interaction between the PVA/Cs matrix and the [TEA-TOS] ionic liquid are evaluated using X-ray diffraction (XRD) and FTIR spectroscopy. XRD analysis showed that crystallinity is reduced from 55.65% for PVA/Cs blend to 29.82% for PVA/Cs/40 wt% TEA-TOS composite. The shifts and changes in the intensity of the FTIR bands of the PVA/Cs blend upon incorporation of [TEA-TOS] showed the formation of strong intermolecular interactions between PVA/Cs and [TEA-TOS]. The energy gap and Urbach energy values, as well as linear/nonlinear optical parameters of the composites are estimated. It is found that the bandgap reduced from 5.62/5.01 eV for pure PVA/Cs to 5.00/4.68 eV for PVA/Cs/40 wt% [TEA-TOS], indicating improved optoelectronic performance. UV–Vis spectroscopy demonstrated confirmed that the optical properties of the samples could be tailored by adjusting the [TEA-TOS] content. The Wemple model further confirmed the modification of electronic transitions, demonstrating that the incorporation of [TEA-TOS] effectively tunes the optical behavior of PVA/Cs films for potential optoelectronic applications.

## Introduction

Polyvinyl alcohol (PVA) has garnered significant attention because of its promising properties in diverse applications^1^. PVA is characterized by a carbon chain containing hydroxyl (OH) groups on carbon atoms bonded to methane. These groups possess an inherent ability to form hydrogen bonds, which significantly influences the composition and properties of PVA-containing polymer blends. Furthermore, PVA offers advantages over other polymers, including superior transparency, flexibility, durability, biocompatibility, biodegradability, and cost-effectiveness. These properties make PVA a suitable choice for a wide range of applications, from optoelectronics to the medical field^2,3^.

Chitosan (Cs) is a semi-crystalline polysaccharide composed of D-glucosamine and N-acetyl-D-glucosamine, is formed by the removal of the acetyl group from chitin^4^. Cs possesses antimicrobial activities with high biocompatibility^5^, is biodegradable, and is also less harmful. The polycationic nature of Cs promotes its interaction with oppositely charged substances, such as proteins, through the ionic bonding^6^. Thanks to its unique properties, such as its abundant natural production, high ability to bind proteins, physiological inertness, smooth facilitation of biological reactions, gel-forming ability, and mechanical strength, chitosan is a suitable choice as a stabilizing polymer among all natural and synthetic polymers^7^. Cs is also a noteworthy material due to its chemical and physical properties, including the formation of microfibers in the dissolved state and solid crystals in the solid state^8^.

Polyvinyl alcohol’s (PVA) ability to blend well with natural polymers makes it a versatile choice in the composite materials industry. Blends created by combining PVA with natural polymers such as chitosan can provide desirable properties for both components^9^. PVA/Cs blends are characterized by high physicochemical and mechanical properties and their high transparency makes them a versatile material suitable for a wide range of applications^10^. PVA/CS also possesses unique properties such as low cost, solubility, and therefore ecofriendly^11,12^. Furthermore, the presence of hydroxyl groups in PVA/CS blend makes it an environmentally friendly material, contributing to the development of sustainable and efficient composite materials.

Tetraethyl ammonium toluene-4-sulfonate ([TEA]^+^[TOS]^−^) ionic liquid electrolyte is basically consisting of tetraethyl ammonium (organic) cation and toluene-4-sulfonate (inorganic) anion and has the chemical form [C_15_H_27_NO_3_S]. It is a water-soluble electrolyte characterized by a high degree of intra -and intermolecular hydrogen bonding. Ionic liquids are characterized by properties such as low vapor pressure, high reactivity with solvents, and good conductivity. It has been widely and successfully used in electrochemistry, not only as a supporting electrolyte in aqueous and non-aqueous solvent systems, but also in various electrochemical methods of industrial and analytical importance^13^.

The innovative properties of polymer composites have attracted considerable interest in their improvement^14^, opening new horizons in optical applications. One of the simplest and most common techniques used in the manufacture of polymer composites is the addition of suitable fillers^15^. The addition of inorganic fillers to polymer blend matrices has attracted widespread scientific interest because it provides an effective route for modifying polymer composites characteristics and with improved optical and electrical properties^16^. The concentration, type, and actual cost of the filler in the host polymer matrix affect the physical and chemical properties of the resulting composite materials, and consequently, their suitability for industrial applications^17^.

It is worth noting that many different optical applications, including integrated optics, broadband optical communication, optical computing, refractive index modulation, and others, rely primarily on the fabrication of materials with high third-order nonlinear optical susceptibility, as well as high nonlinear refractive indices. Therefore, many studies and research have currently focused on the nonlinear optical (NLO) properties of materials such as organic and inorganic materials^18^, nanomaterials^19,20^, polymeric nanocomposites^21,22^, and others.

In the literature there are many studies concerning the synthesis of PVA/Cs/IL composites and most of them for investigation the electrical conductivity^23–26^, antibacterial activity^27–29^ and water desalination^30^. To our knowledge, no previous studies have addressed the incorporation of [TEA][TOS] into PVA/Cs blend. This gap presents a new opportunity for investigating the effect of [TEA][TOS] on the physical and chemical properties of PVA/Cs blend. Furthermore, the thermal, optical, and electrical properties of PVA/Cs/[TEA][TOS] composites have not been comprehensively studied. Therefore, this study aims to fill this research gap by giving new insights into the effect of [TEA][TOS] incorporation and evaluating the potential of PVA/Cs/[TEA][TOS]composites for optoelectronic applications. XRD and FTIR analyses confirmed the successful synthesis of PVA/Cs/[TEA-TOS] composites. The structure, TGA and linear/nonlinear optical properties of PVA/Cs were also investigated as a function of the [TEA-TOS] filler ratio. This work showed that flexible PVA/CS/[TEA-TOS] composite is successfully prepared and their linear/nonlinear characteristics were improved for use in optoelectronics devices.

## Experimental work

### Materials

Chitosan with a molecular weight of ~ 300,000 and N-deacetylation degree of 86% is supplied by Acros Organics (USA). PVA (C_2_H_4_O)_n_ is purchased from laboratory Rasayan (India) with a molecular weight of ~ 16,000 and Tetraethylammonium toluene-4-sulfonate [TEA][TOS] is supplied by Fluka (Germany) Fluka with molecular weight of 301.44 and purity > 98%.

### Preparation method

#### Preparation of PVA/Cs blend

PVA (0.5 g) is dissolved in 50 mL of distilled water at 353 K under magnetic stirring for 6 h until a clear solution is obtained and 0.5 g of chitosan (Cs) is dissolved in a solvent containing 40 mL of distilled water and 10 mL of acetic acid at 313 K with continuous magnetic stirring for 5 h to obtain a transparent solution. This ratio of PVA and Cs (50/50 by weight) is chosen to ensure a homogeneous and compatible mixture. The prepared PVA and Cs solutions are then mixed together under continuous mechanical stirring for 2h to obtain a homogeneous mixture. The mixture is then poured into glass dishes in an oven at 333 K for 72 h. Following complete evaporation of the solvent, homogeneous and flexible films of PVA/Cs are successfully obtained.

#### Preparation of PVA/Cs/[TEA-TOS] composites

**Scheme 1:**
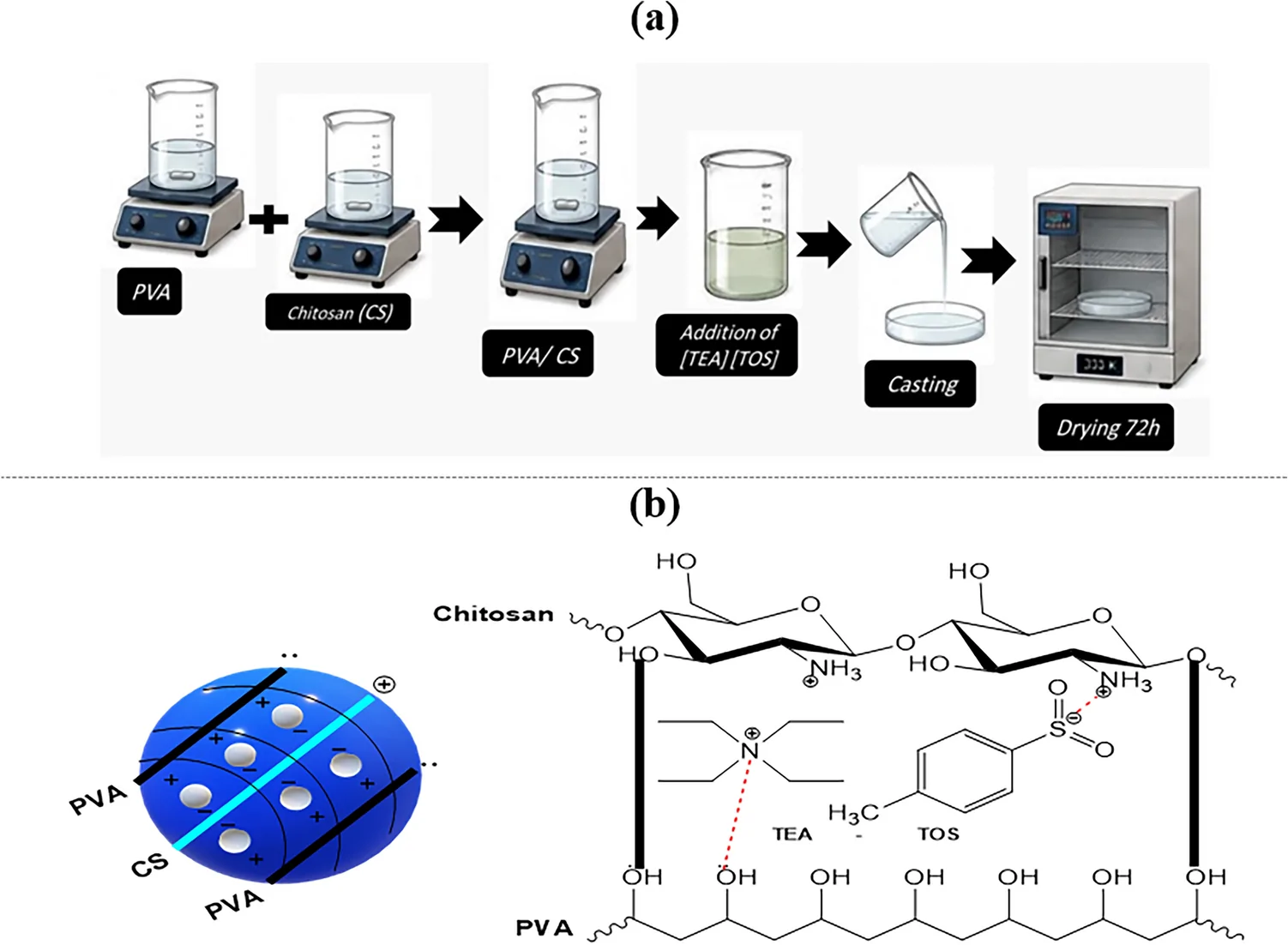
(**a**) The procedure of preparation method and (**b**) the interaction between the components of PVA/CS/[TEA-TOS] composite.

[TEA-TOS] was added at varying concentrations, ranging from 10 to 40 wt%, into a PVA/Cs solution dissolved in 100 mL of distilled water containing 1% acetic acid under magnetic stirring for 1 h to ensure complete homogeneity. Free-standing films of PVA/Cs/[TEA-TOS] are prepared by pouring the solution into Petri dishes in an oven at 333 K for 72 h to remove any residual solvent. The procedure of preparation method and interaction mechanism between PVA/Cs and [TEA-TOS] is shown in Scheme 1a–b.

The PVA/CS/[TEA-TOS] composite is formed according to Scheme 1, as the two polymers of PVA and chitosan interact by hydrogen bonding as well as dipole–dipole interaction based on the variety in electronegativity. On the other hand, the embedded tetraethylammonium toluene-4-sulfonate is an ionic salt with a dual-charged dipole that can react via electrostatic interaction. As the positively charged ammonium salt electrostatically interacts with the highly electronegative hydroxyl groups (oxygen carries two lone pairs of electrons). Additionally, the negatively charged toluene-4-sulfonate anion is attracted towards the oppositely charged protonated chitosan.

### Characterization techniques

XRD measurements of all samples are carried out by using Philips PW 1390 X-ray diffractometer with a monochromator beam of CuK_α_ radiation with λ = 1.5406 Å (I = 40 mA and V = 40 kV). The 2θ angle is scanned at speed of 2θ = 2°/min in the range of 5° to 70°. FTIR spectra of all samples are recorded at room temperature in the wavenumber range 4000–400 cm^−1^ using (IR) Spectrometer (MATTSON 5000 FTIR). Thermal degradation of all samples are monitored in the temperature range from RT to 800 °C with heating rate of 10 °C/min using Netzsch TG 209 F1 Libra Thermogravimetric analyzer (TGA) under nitrogen atmosphere. An appropriate amount of the sample is loaded into alumina (Al_2_O_3_) crucibles, while nitrogen gas is supplied at a constant flow rate of 20 mL/min. UV/Vis spectra are measured in the range from 200 to 800 nm by UV–Vis spectrophotometer (UV-1601 PC, Shimadzu, Japan).

## Result and discussion

### XRD

**Fig. 1:**
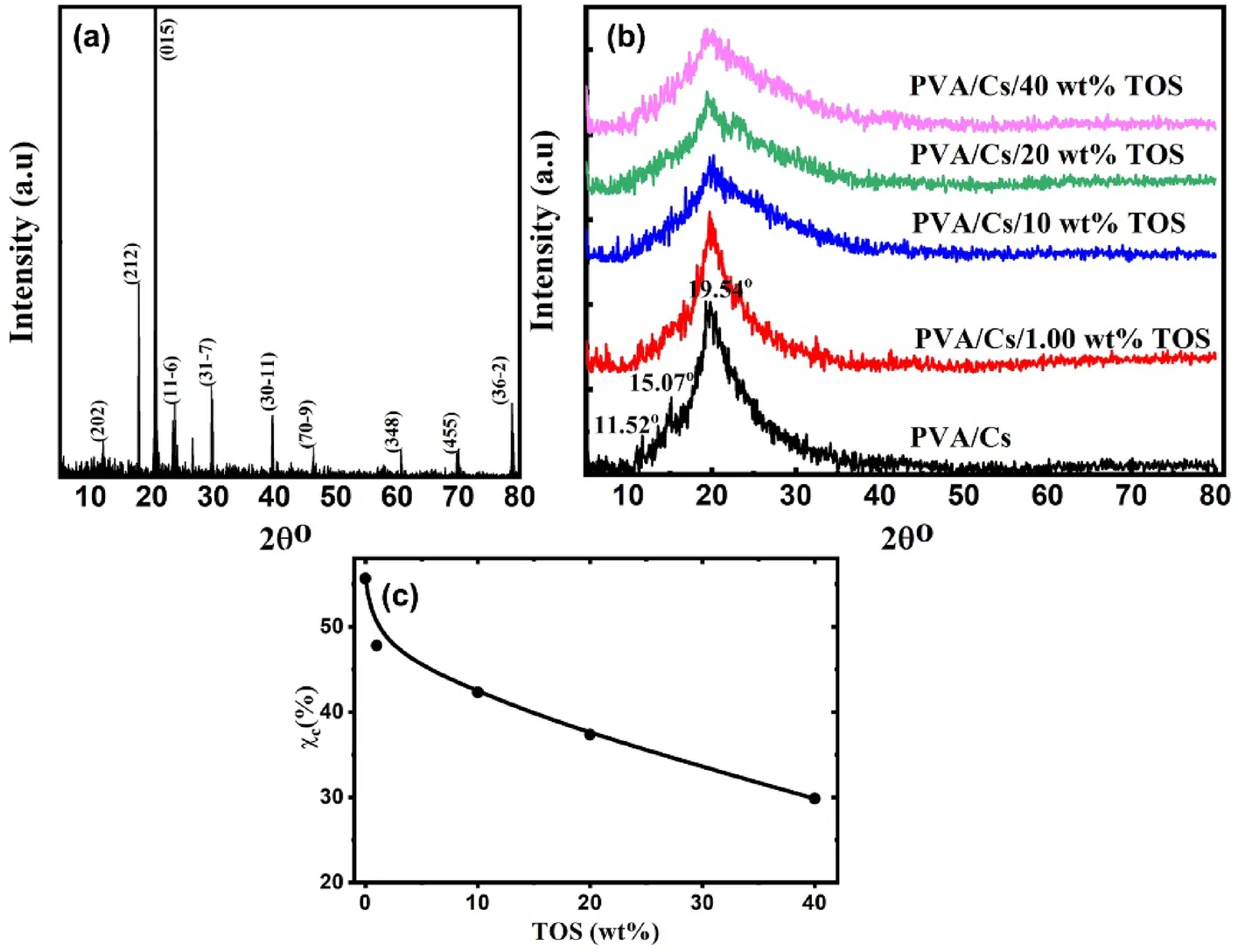
XRD of (**a**) pure TEA-TOS, (**b**) PVA/Cs/TEA-TOS composites and (**c**) χ_c_ versus TOS wt%.

Figure 1a presents XRD pattern of pure [TEA-TOS], which shows that it is a highly crystalline material, with several sharp peaks at the angles 2θ = 17.92°, 20.79°, 23.85°, 29.79°, 17.92°, 39.68°, 46.38°, 60.72°, 70.07°, 78.70° respectively. The corresponding planes of these diffraction angles are inserted in Fig. 1a. On the other side, Fig. 1b shows the XRD patterns of PVA/Cs blend and its composites with different TOS loading ratio. The pure PVA/Cs blend showed three diffraction angles at 2θ = 11.52°, 15.07° and 19.54° indicating the semicrystalline nature PVA/Cs blend^2,11^. The diffraction angle at 2θ = 11.52° is related to the hydrated crystalline structure^31^. As the TOS content is increased, the diffraction angles at 2θ = 11.52° and 15.07° in the composite samples nearly disappeared.

Also, the peak at 2θ = 19.51° shifted slightly to 2θ = 20.07° and its intensity decreased and become more broad. These obvious changes indicate that with an increase in the TOS content, the structure of PVA/Cs blend is changed, i.e., crystallinity has been decreased while amorphousness is increased. The degree of crystallinity of PVA/Cs/TEA-TOS composites is estimated using the following relation^32^:

1a $$\chi_{c} = \frac{{{\mathrm{A}}_{{\mathrm{c}}} }}{{{\mathrm{A}}_{{\mathrm{c}}} + {\mathrm{A}}_{{\mathrm{a}}} }} \times 100$$

where A_c_ and A_a_ correspond to the integrated areas under the crystalline and amorphous peaks, respectively. This approach is widely applied for semicrystalline polymer to estimate the crystallinity degree within the polymer matrix. The crystallinity degree (χ_c_) is found to decrease substantially from 55.65% for the pure PVA/Cs blend to 29.82% for the PVA/Cs/40 wt% TEA-TOS composite, as demonstrated in Fig. 1c. The observed reduction suggests that the incorporation of the TEA-TOS disrupts the ordered packing of polymer blend chains, thus reducing the overall crystallinity. Similar behavior is reported for chitosan when doped with [BMIM][TfO], the crystallinity degree is reduced from 58.58% for the pure Cs blend to 23.01% for the Cs/50 wt% [BMIM][TfO]composite^26^. This reduction in the crystallinity of the composite samples indicated that [TEA-TOS] acts as a plasticizing and amorphizing agent^33^.

The crystallite size is estimated by using Debye–Scherrer’s equation, as follow:

1b $$D = {\raise0.7ex\hbox{${0.94\lambda }$} \!\mathord{\left/ {\vphantom {{0.94\lambda } {\beta \cos \theta }}}\right.\kern-0pt} \!\lower0.7ex\hbox{${\beta \cos \theta }$}}$$

where, λ, β and θ are the wavelength of X-ray, the full width at half maximum (FWHM) in radians and the diffraction angle. It is found that the average mean crystallite size of [TEA-TOS] is in the range of 98.58 nm.

### FTIR spectroscopy

**Fig. 2:**
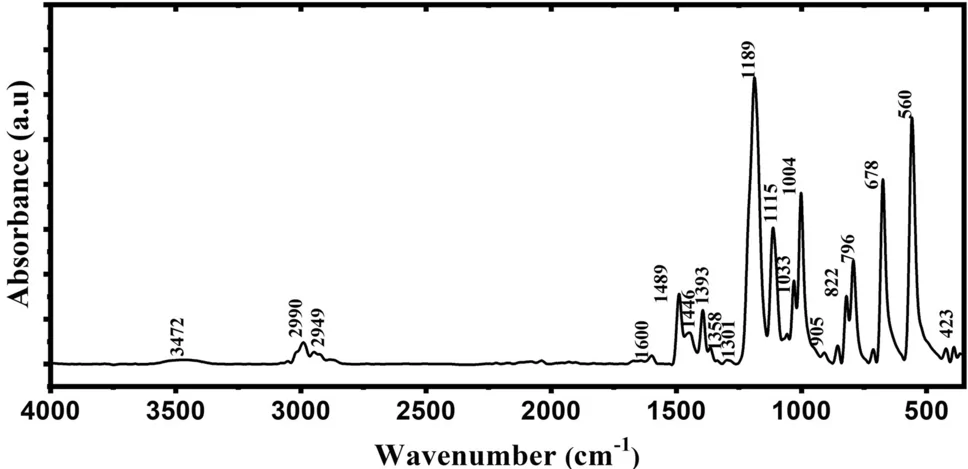
FTIR spectrum of TEA-TOS.

Figure 2 demonstrates FTIR of Tetraethylammonium toluene-4-sulfonate. The small broad centered at 3472 cm^−1^ is attributed to the O–H stretching vibration, while the absorption peaks at 2990 cm^−1^ and 2949 cm^−1^ are characteristic of alkyl C–H stretching and C–H asymmetric stretching vibration^34,35^. The absorption peaks between 1600 and 1500 cm^−1^ are ascribed to the ring stretching vibrations of the benzene ring in the toluene-4-sulfonate anion. The absorption peaks at 1489 cm^−1^ and 1446 cm^−1^ are ascribed to the aromatic C=C Stretching from the toluene ring and to asymmetric CH_2_ bending vibration^36,37^. The absorption peaks at 1358 cm^−1^ and 1301 cm^−1^ are assigned to the aromatic amine C–N stretching and to the twisting mode of CH_2_^38^. The absorption peaks at 1189 cm^−1^ and 1066 cm^−1^ are characteristics of CH_3_ rocking mode vibrations in ethyl group while the peak at 1033 cm^−1^ is attributed C–N asymmetric stretching vibration^36^. The bands between 1190 to 1030 cm^−1^ are asymmetric and symmetric stretching vibrations of the SO_2_ group. The absorption peaks at 1004 cm^−1^ and 905 cm^−1^ are attributed to the rocking mode of CH_3_ group and to symmetric stretching vibrations of C–C bond which is observed as a weak peak^38^. The absorption peak at 822 cm^−1^ is assigned to Out-of-plane C–H bending for para-substituted benzene ring. The peaks at 796 cm^−1^ is characteristics of CH_2_ rocking mode vibration, at 560 cm^−1^ is assigned to torsional N–H oscillations and at 423 cm^−1^ is characteristic to the skeletal vibration of the ethyl groups^36,37^.

**Fig. 3:**
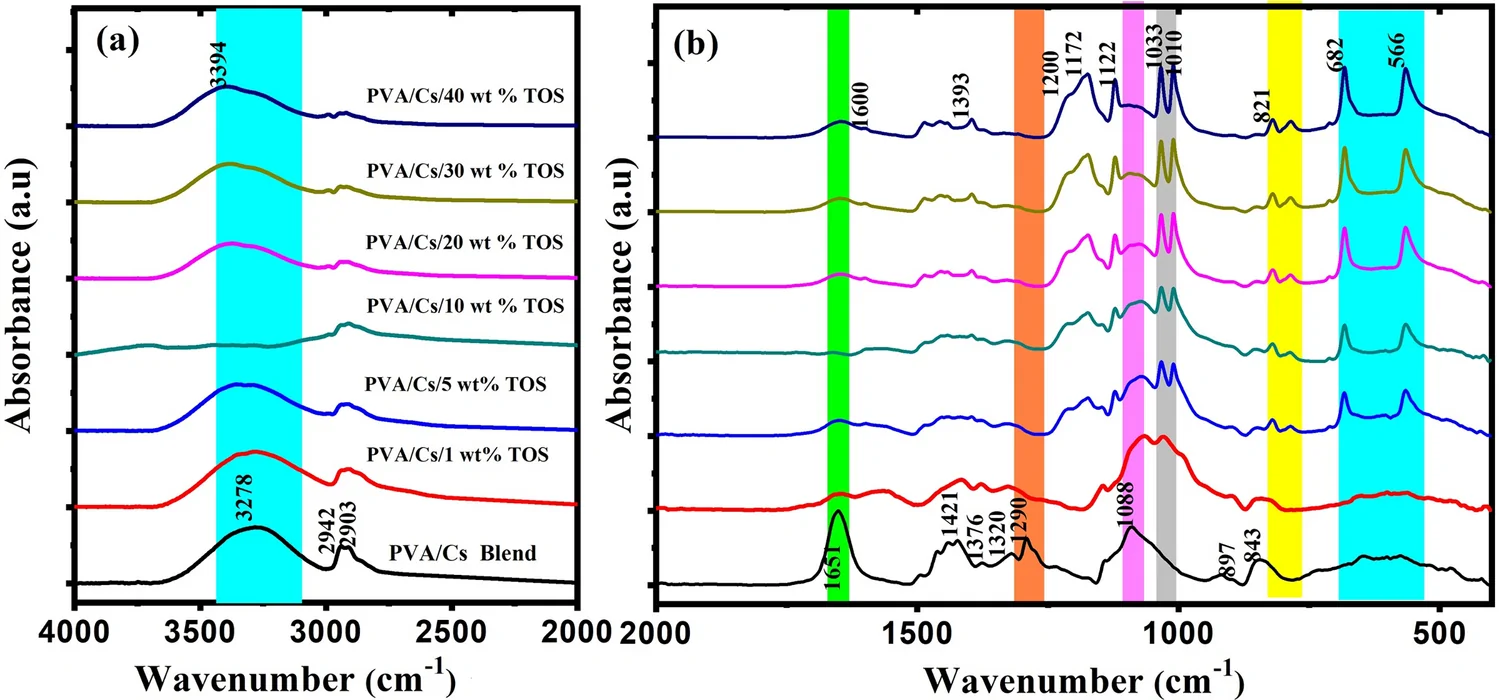
FTIR spectra of PVA/Cs/TEA-TOS in the range of (**a**) 4000–2000 cm^−1^ and (**b**) 2000–400 cm^−1^.

Figure 3a illustrates FTIR spectra of pure PVA/Cs blend and PVA/Cs/TOS composites in the range 4000–2000 cm^−1^ and Fig. 3b in the range 2000–400 cm^−1^. The spectrum of pure PVA/Cs bled showed characteristic absorption peaks of PVA and Cs. The broad band at 3278 cm^−1^ is due to the N–H stretching vibration of primary amino groups overlapping with O–H stretching vibration. Because this peak is not sharp, the hydroxyl groups are linked through intramolecular and intermolecular hydrogen bonding^9,38^. The peaks at 2942 and 2903 cm^−1^ represent the asymmetric and symmetric stretching vibrations of CH_2_ and are attributed to the pyranose ring^39^. The peak at 1651 cm^−1^ is assigned to the carbonyl C=O stretching of the amide group (amide I)^40^. The absorption peak at 1421 cm^−1^ is ascribed to OH bending vibrations of the hydroxyl groups^39^. The absorption peak at 1376 cm^−1^ represents the C–H bond vibrations of the methyl group present in the amide group^41^. The peak found at 1320 cm^−1^, which is attributed to the presence of C–N stretching and N–H in the plane bending of the amide groups, confirmed the presence of the amide III band^42^. The peaks at 1291 and 1236 cm^−1^ are attributed to C–H bending and to C–H stretching vibration in rings^43^. The weak shoulder at 1247 cm^−1^ is attributed to O–H bending vibrations. The absorption peak at 1136 cm^−1^ which is observed as a weak shoulder is ascribed to β (1–4) glucosidic stretching^11,44^. The peaks at 1088, 897 and 843 cm^−1^ are attributed to C–O stretching, wagging of C–H bond (bending out of the plane of the monosaccharide ring) and to the C–C stretching vibration, respectively^45–47^.

FTR spectra allow for the identification of differences in the molecular structure of the investigated materials. Based on these spectra and the appropriate functional groups, the chemical interactions between PVA/Cs and TOS in the polymer matrix synthesis process can be monitored. The hydroxyl, amine and amide groups are the most reactive sites in the PVA/Cs blend matrix and participate in reactions with surrounding, anions and cations^48,49^. After TOS doping, several changes are observed in the characteristic bands of the PVA/Cs blend matrix in the FTIR spectra, as shown in Fig. 3. These changes suggest possible interactions between these entities. For PVA/Cs/TOS composites, the band observed at 3278 cm^−1^ in the spectrum of pure PVA/Cs is blue shifted to 3394 cm^−1^ with shift order of 116 cm^−1^ for PVA/Cs/40 wt% TOS composite, indicating a strong hydrogen bond interaction between blend matrix and the filler functional group^50^. The bands of CH_2_ at 2942 and 2903 cm^−1^ are also observed to be blue shifted to higher wavenumber to 2948 and 2921 cm^−1^ with shift order of 6 and 18 cm^−1^, respectively. On the other hand, the bands of amide I, OH bending and C–O stretching at 1651 cm^−1^, 1421 cm^−1^ and 1088 cm^−1^ are observed to be red shifted to lower wavenumber at 1643 cm^−1^, 1395 cm^−1^ and 1075 cm^−1^, with shift order of 8, 26 and 13 cm^−1^, respectively, while the band of amide III at 1320 cm^−1^ has been blue shifted to1334 cm^−1^ with shift order of 14 cm^−1^ and the band of C–H bending at 1291 cm^−1^ is disappeared. The wavenumber and assignment of the characteristic bands are given in Table 1. The most interesting thing, the intensity of the bands at 1033, 1010, 682 and 566 cm^−1^ are increased with increasing TOS content.

**Table 1:** FT-IR spectra characteristics of PVA/Cs/TEA-TOS composites.

Wavenumber (cm^−1^)		Assignment	References
PVA/Cs	PVA/Cs/40wt% TOS		
3278	3394	stretching vibration of O–H and N–H group, blue shift	Fahmy et al.^9^, Kumirska et al.^39^
2942	2948	asymmetric stretching vibration of CH_2_, blue shift	Bujňáková et al.^40^
2903	2921	symmetric stretching vibration of CH_2_	Bujňáková et al.^40^
1651	1643	C=O stretching (amide I), red shift	Zarif et al.^41^
1421	1395	OH bending vibration, red shift	Bujňáková et al.^40^
1376		C–H vibrations of the methyl group	Bujňáková et al.^40^
1320	1334	C–N stretching and N–H in the plane bending (amide III), blue shift	Staroszczyk et al.^42^
1291		C–H bending	Gieroba et al.^43^
1247		O–H bending vibrations	
1235		C–H stretching vibration in rings	Gieroba et al.^43^
1136		β(1–4) glucosidic stretching	Abdelfattah et al.^11^, Ibupoto et al.^44^
1088	1075	C–O stretching vibration, red shift	Choo et al.^45^, Visan et al.^46^, Bonilla et al.^47^
897		wagging of C–H bond	Choo et al.^45^, Visan et al.^46^, Bonilla et al.^47^
843		C–C stretching vibration	Choo et al.^45^, Visan et al.^46^, Bonilla et al.^47^

### Peak fitting analysis of FTIR spectra of PVA/Cs/TOS composites

**Fig. 4:**
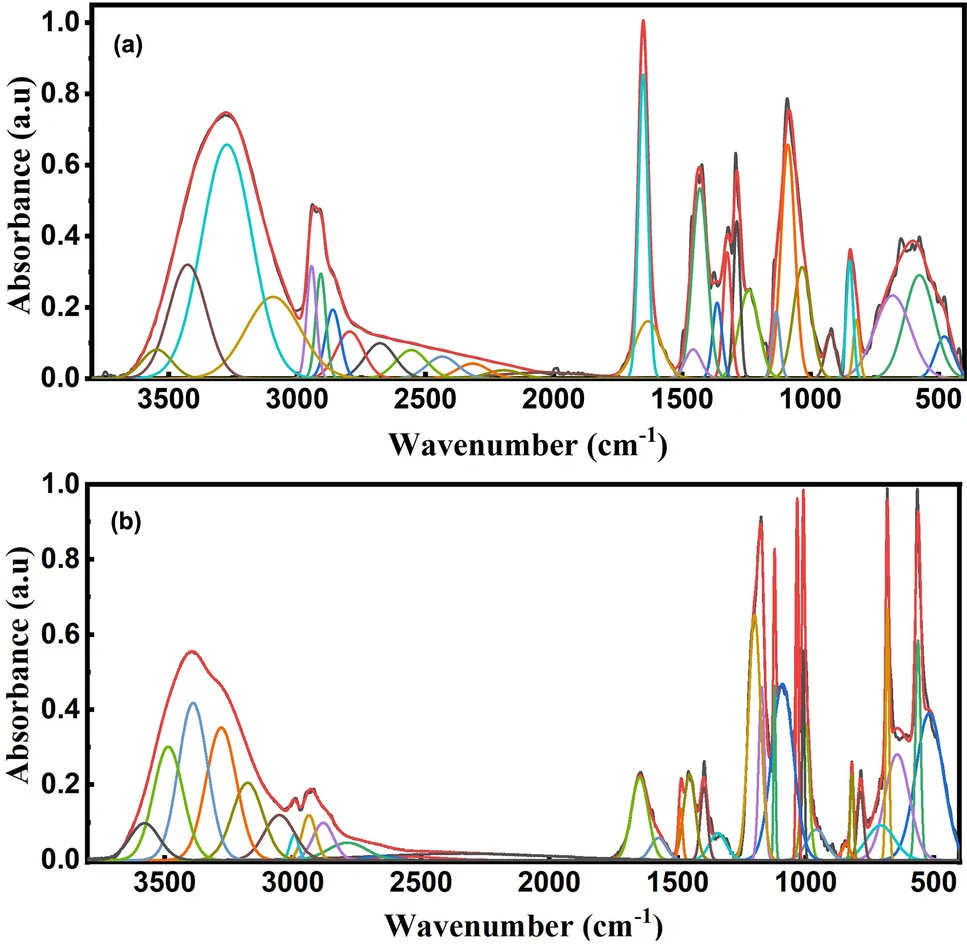
The deconvolution analysis of (**a**) PVA/Cs and (**b**) PVA/Cs/40wt% TEA-TOS.

The deconvolution analysis of FTIR is performed for all samples to calculate the area under peak. The infrared spectrum is examined and quantitatively stimulated using Gaussian functions, employing the standard curve fitting software [Peak Fit 4.12], which automatically adjusts the peak position for each band (intensity and width) to give the best fit. The deconvolution analysis of FTIR of pure PVA/Cs blend and PVA/Cs/40wt% TOS composite as a representative sample for the composites is shown in Fig. 4a, b.

**Fig. 5:**
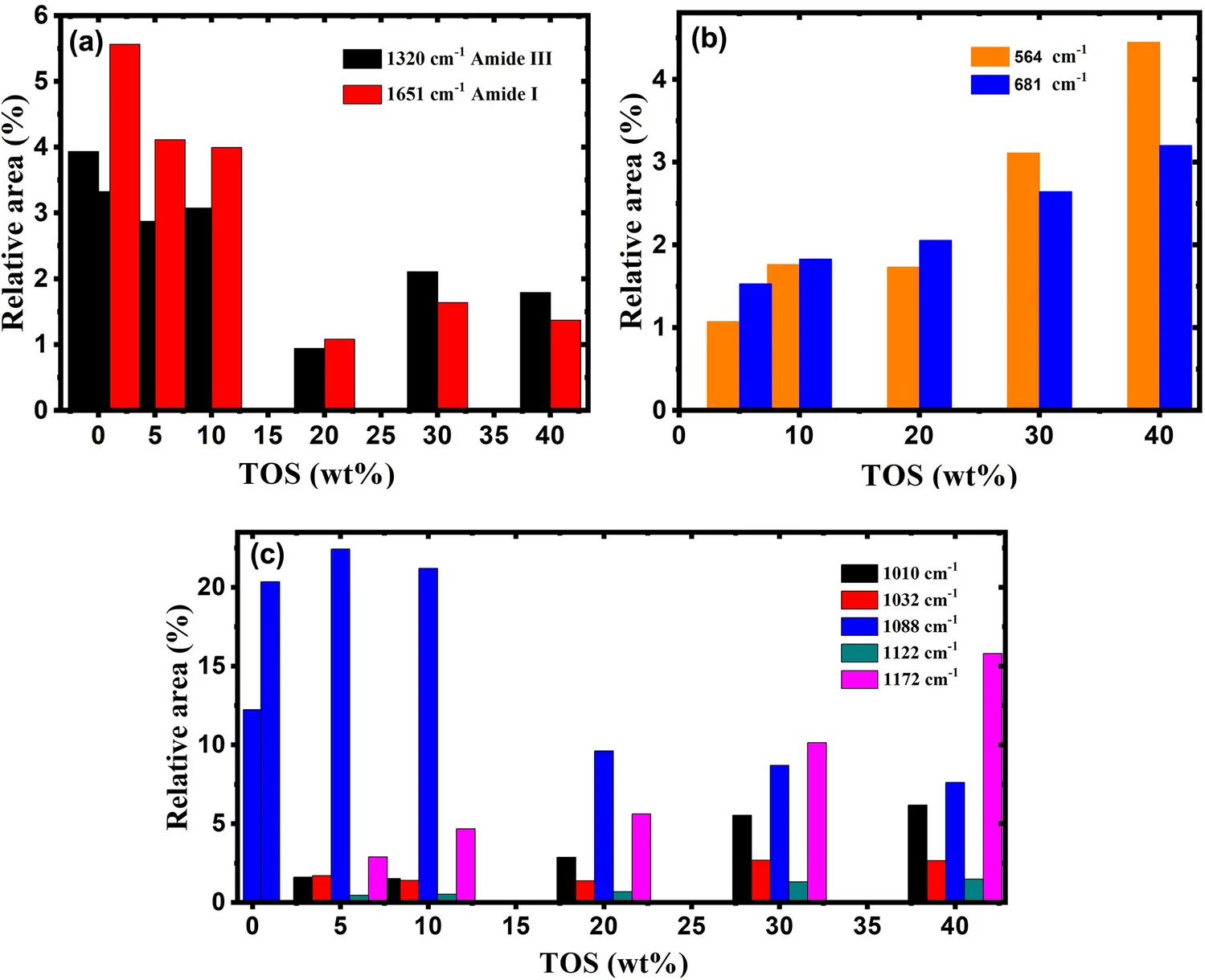
The relative area of characteristic peaks versus TOS content. (**a**) at the bands 1561 cm^-1^ and 1320 cm^-1^, (**b**) at the bands 681cm^-1^ and 564 cm^-1^ and (**c**) at the bands 1172, 1122, 1088, 1032 and 1010 cm^-1^.

Figure 5a–c displays the change in the relative areas of some characteristic bands as a function of TOS content. A consistent decrease in the relative areas of both the Amide I and Amide III bands is observed with increasing TOS content, as shown in Fig. 5a. This decrease in relative area is attributed to the breaking of C=O, C–N, and N–H bonds. This interpretation is supported by the corresponding decrease in band intensity observed in Fig. 3. On the other hand, the relative area of ​​the two bands at 564 cm^−1^ and 681 cm^−1^ increase with increasing TOS content, as shown in Fig. 5b. Furthermore, the relative area of ​​all bands in the 1172–1010 cm^−1^ range also increases with increasing TOS content, except for the band at 1010 cm^−1^, as shown in Fig. 5c. This band, as is known, is responsible for the crystallinity of PVA, and a decrease in its relative area or intensity indicates a decrease in the crystallinity of the PVA/Cs matrix. This result is in agreement with the XRD analysis, which exhibited a reduction in the crystallinity degree of the PVA/Cs blend as the [TEA-TOS] content increased. This conclusion is confirmed by the change in Urbach energy with increasing TOS content, as will be discussed later in the UV section.

### TGA/DTG

**Fig. 6:**
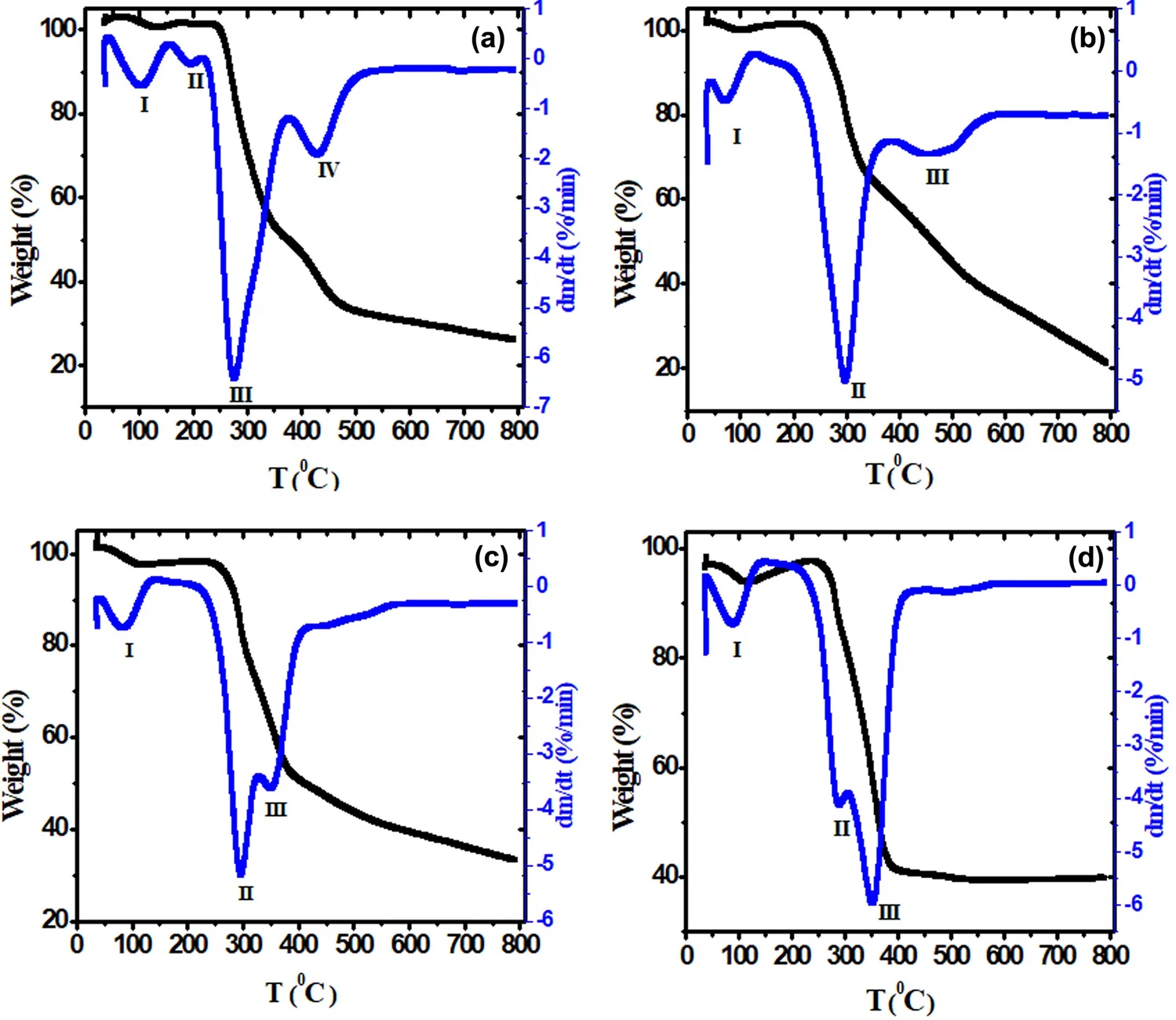
The TGA/DTG of (**a**) PVA/Cs, (**b**) PVA/Cs/10 wt%TEA-TOS, (**c**) PVA/Cs/20 wt% TEA-TOS and (**d**) PVA/Cs/40 wt% TEA-TOS.

Figure 6 shows the TGA/DTG plots of pure PVA/Cs blend and PVA/Cs/TOS composites with different loadings of TOS over a temperature range from RT to 800 °C. It is observed that the decomposition of the PVA/Cs blends occurred in four stages, while the decomposition of the PVA/Cs/TOS composites occurred in three stages. Table 2 shows the weight loss values ​​and the maximum temperature (T_m_) for each decomposition stage for all samples, as well as the initial decomposition temperature (T_i_) and residual weight. For the pure PVA/Cs blend, the weight loss in the first and second stages is attributed to the water loss. Considering the structure of the PVA/Cs blend, it was observed that water molecules are able to bind to the hydroxyl and amine polar groups. It has been previously reported that the interaction between water molecule and hydroxyl group is stronger than with amine group^51^. It was also found that the relative amount of water molecules bound to these two polar groups, changes during the process of water absorption. The main degradation process is carried out in the third stage with a weight loss of 49.53% at a maximum temperature of 277.10 °C. The second stage corresponds to the decomposition (oxidative and thermal) of PVA/Cs, the removal of volatile products, and evaporation. In general, the thermal decomposition of polysaccharides begins with random cleavage of glycosidic bonds, followed by further decomposition to form butyric and acetic acids, as well as a series of lower fatty acids^52,53^. Moreover, the weight loss in the fourth stage may be related to the cleavage of the C–C bonds in the PVA/Cs chains^54^. It is observed that both the initial temperature and residual mass of PVA/Cs/TOS composites increased with increasing TOS content, indicating that the composite samples are more thermally stable than pure PVA/Cs blends. Other researchers have previously reported the same behavior^55,56^.

**Table 2:** Maximum temperature, (*T*_*m*_) and weight loss (%) for PVA/Cs/[TEA][TOS] composites.

Sample	Stage I		Stage II		Stage III		Stage IV		Residual mass	T_i_ (°C)
	*T*_*m*_ °C	Weight (%)	*T*_*m*_ °C	Weight (%)	*T*_*m*_ °C	Weight (%)	*T*_*m*_ °C	Weight (%)		
PVA/Cs	102.9	1.35	197.95	1.22	277.10	49.53	431.17	19.55	25.66	242.62
PVA/Cs/10 wt% TEA-TOS	71.10	1.05	299.82	34.78	464.16	22.06	–	–	21.29	244.12
PVA/Cs/20 wt% TEA-TOS	84.13	2.18	296.91	25.47	352.02	24.02	–	–	33.32	254.69
PVA/Cs/40 wt% TEA-TOS	88.83	2.53	291.06	18.14	352.69	40.18	–	–	39.06	262.29

### Thermal decomposition kinetics

The thermal decomposition kinetics can be estimated using thermogravimetric data, keeping in mind that the decomposition rate depends only on two parameters, temperature, (T), and the degree of conversion, (g).

2 $$\frac{dg(t)}{{dt}} = \beta \frac{dg}{{dT}} = k(T)f(g)$$

where β is the rate of heating and the degree of conversion, (g) is defined as follows

3 $$g = \frac{{w_{i} - w_{t} }}{{w_{i} - w_{f} }}$$

where, *w*_*i*_ is the initial weight, *w*_*t*_ is the weight at certain time and *w*_*f*_ is the final weight at the end of reaction, respectively. Values of *g* reflect the overall transformation progress which can include multiple stages, each with a specific extent of transformation and *f*(*g*) is the kinetic reaction model. In the case of a thermally stimulated process, the rate constant, k(T), is usually expressed by the Arrhenius equation as follows:

4 $$\frac{dg(t)}{{dt}} = f\exp \left( { - \frac{{E_{a} }}{RT}} \right)f(g)$$

where *f* is the frequency factor, *E*_*a*_ is the activation energy and *R* is the gas constant (8.314 J. mol^−1^ K^−1^), respectively. The relationship between frequency factor (*f*) and entropy activation (ΔS) can be written as follows:^57^:

5 $$f = \frac{{e\gamma k_{B} T_{m} }}{h}\exp \left( {\frac{\Delta S}{R}} \right)$$

where, e is the Neper number (e = 2.7183), γ is the transmission coefficient (unity for monomolecular reaction), *h* is the Planck’s constant and* k*_*B*_ is the Boltzmann’s constant, respectively. Thus, the rate constant *k*(T) can be expressed as follows:

6 $$k(T) = \frac{{\gamma ek_{B} T{}_{m}}}{h}\exp \left( {\frac{\Delta S}{R}} \right)\exp \left( { - \frac{{E_{a} }}{RT}} \right)$$

Hence, Δ*S* can be estimated using Eq. (5) as follows:

7 $$\Delta S = 2.303\,R\log \left( {\frac{fh}{{e\gamma k_{B} T_{m} }}} \right)$$

and the activation energy (*E*_*a*_) is defined as follows:

8 $$E_{a}^{{}} = \Delta H + RT_{m}$$

where ΔH is the activation of enthalpy and then the Gibbs free energy (ΔG) is calculated using the following equation:

9 $$\Delta G\,\, = \,\Delta H\, - \,T_{m} \,\Delta S\,$$

The values of *E*_*a*_, *ΔS*, *ΔH* and *ΔG* of pure PVA/Cs blend and PVA/Cs/TOS composites can be evaluated by employing Coats-Redfern model, using the reaction order n = 1, according to the following equation^58^

10 $$\log \left[ { - \frac{{\log \left( {1 - g} \right)}}{{T^{2} }}} \right] = \log \frac{fR}{{\beta E_{a} }}\left( {1 - \frac{2RT}{{E_{a}^{{}} }}} \right) - \frac{{E_{a} }}{2.303R}\frac{1}{T}$$

In case of $2RT/E_{a}$ <  < 1, the Eq. (10) will become^58^

11 $$\log \left[ { - \frac{{\log \left( {1 - g} \right)}}{{T^{2} }}} \right] = \log \frac{fR}{{\beta E_{a} }} - \frac{{E_{a} }}{2.303R}\frac{1}{T}$$

**Fig. 7:**
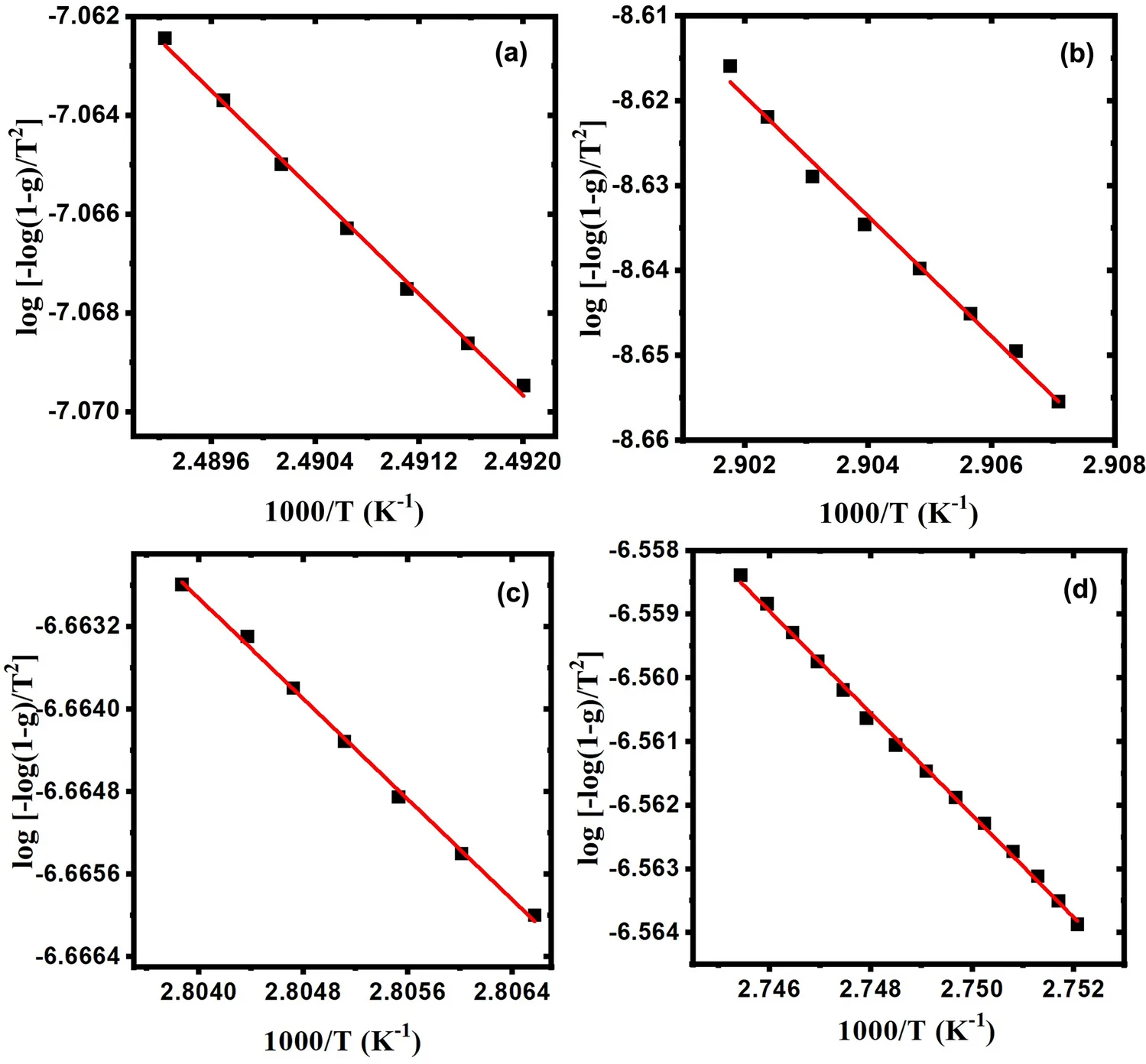
Log [-log(1-g)/T^2^] against 1/T of (**a**) PVA/Cs, (**b**) PVA/Cs/10wt% TOS, (**c**) PVA/Cs/20wt% TOS and (**d**) PVA/Cs/40wt% TOS.

Figure 7 illustrates the variation of log[−log(1-g)/T^2^] against 1/T for pure PVA/Cs blend and PVA/Cs/TOS composite samples in the first stage of thermal degradation. The intercept [log (*fR/βE*_*a*_)] and slope [−*E*_*a*_*/2.303R*] of the fitted curves as shown in Fig. 7 are used to calculate the values of *E*_*a*_ and *f*. The values of *E*_*a*_, *f*, *ΔS*, *ΔH* and *ΔG* of the samples in all decomposition stages are estimated and summarized in Table 3.

**Table 3:** The kinetic parameters for PVA/Cs and PVA/Cs/TEA-TOS based on C–R approach.

Sample	*E*_*a*_ (kJ/mol)	R^2^	*F *(Hz)	Δ*S* (kJ/mol K)	Δ*H* (kJ/mol)	Δ*G* (kJ/mol)
Stage I						
Pure PVA/Cs	49.208	0.9967	427.23	− 0.193	46.071	119.19
PVA/Cs/10wt% TEA-TOS	135.75	0.9914	4.52 × 10^15^	0.056	132.88	113.52
PVA/Cs/20wt% TEA-TOS	23.168	0.9975	0.521	− 0.249	20.191	109.57
PVA/Cs/40wt% TEA-TOS	15.317	0.9979	0.027	− 0.274	12.295	112.18
Stage II						
Pure PVA/Cs	37.298	0.9974	0.9938	− 0.249	33.405	150.05
PVA/Cs/10wt% TEA-TOS	110.47	0.9987	5.19 × 10^07^	− 0.101	105.73	163.58
PVA/Cs/20wt% TEA-TOS	159.49	0.9985	1.24 × 10^15^	− 0.018	154.74	165.40
PVA/Cs/40wt% TEA-TOS	196.25	0.9994	5.31 × 10^15^	0.049	191.55	163.76
Stage III						
Pure PVA/Cs	29.486	0.9920	2.05 × 10^15^	− 0.127	24.916	95.172
PVA/Cs/10wt% TEA-TOS	32.167	0.9980	0.4365	− 0.259	26.024	217.69
PVA/Cs/20wt% TEA-TOS	37.145	0.9981	1.8730	− 0.247	31.929	186.94
PVA/Cs/40wt% TEA-TOS	70.056	0.9981	7.21 × 10^03^	− 0.183	68.716	208.62
Stage IV						
Pure PVA/Cs	27.954	0.9962	0.191	− 0.271	22.108	212.98

It is observed that, the values of *E*_*a*_ are increased from (37.298/29.486) kJ/mol for pure PVA/Cs to (196.25/70.056) kJ/mol for PVA/Cs/40wt% TOS in the second and third stages of degradation, respectively. Our results are in good agreement with previously reported data in the literature^59,60^. Hao et al. reported that the activation energy of chitosan in the second stage ranged from 98.45 to 138.96 kJ/mol, while Stephy et al. showed that activation energy of chitosan/PA blend based on different models is changed from 38.09 to 184.24 kJ/mol. Also it is reported that activation energy of PVA/Cs/ZnO nanocomposites in the different stages is ranged from 23.22 to 89.74 kJ/mol^11^. The slight difference between the published data and our results may be due to various factors such as the heating rate and different gas flow.

**Fig. 8:**
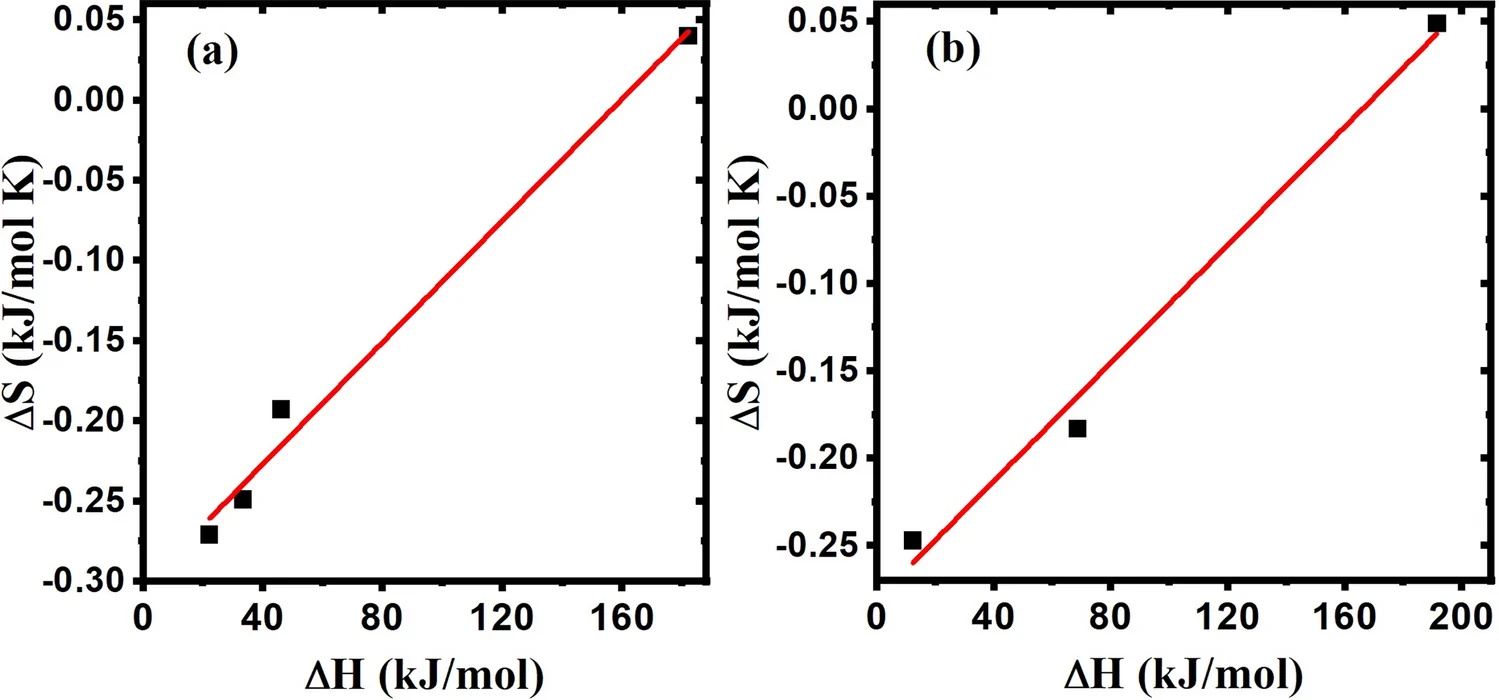
ΔS versus ΔH of (**a**) PVA/Cs and (**b**) PVA/Cs/40wt% TEA-TOS.

**Fig. 9:**
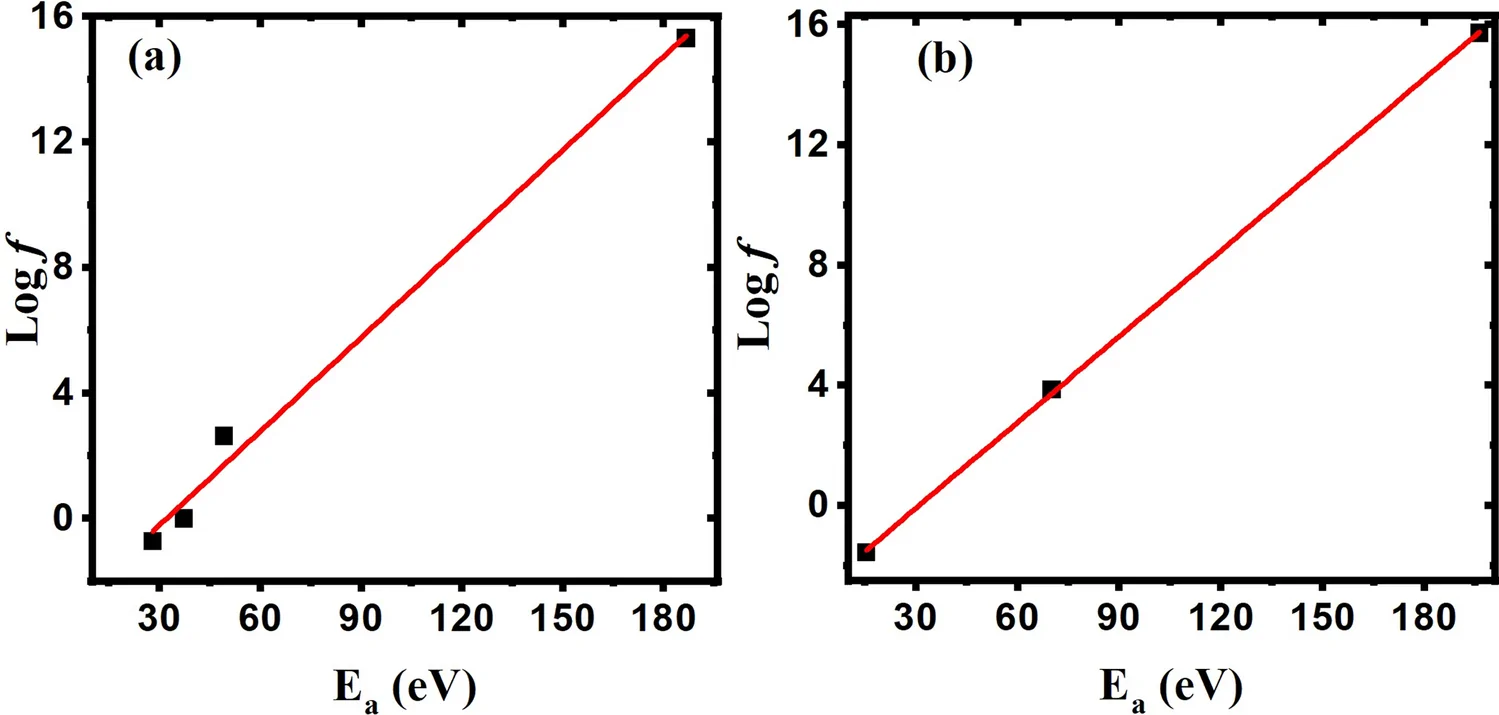
log *f* versus E_a_ of (**a**) PVA/Cs and (**b**) PVA/Cs/40wt% TEA-TOS.

The observed linearity between (*∆S*) and (*∆H*) or the correlation between activation energy (*E*_*a*_) and frequency factor (*f*), confirmed the presence of isokinetic effect or the compensation phenomenon in the PVA/Cs/TOS composites, as shown in Figs. 8a, b and 9a, b^61^. Such behavior is published previously for several polymer blends or polymer composites^15,62,63^. Generally, when the temperature of a substance increases in order to reach equilibrium, structural changes may occur within the substance. These changes may be the reason for the linear relationship between *ΔH* and *ΔS*, meaning that the variation in the values ​​of ΔH is compensated for by the variation in the values ​​of ΔS^64^.

### UV/Vis spectroscopy

**Fig. 10:**
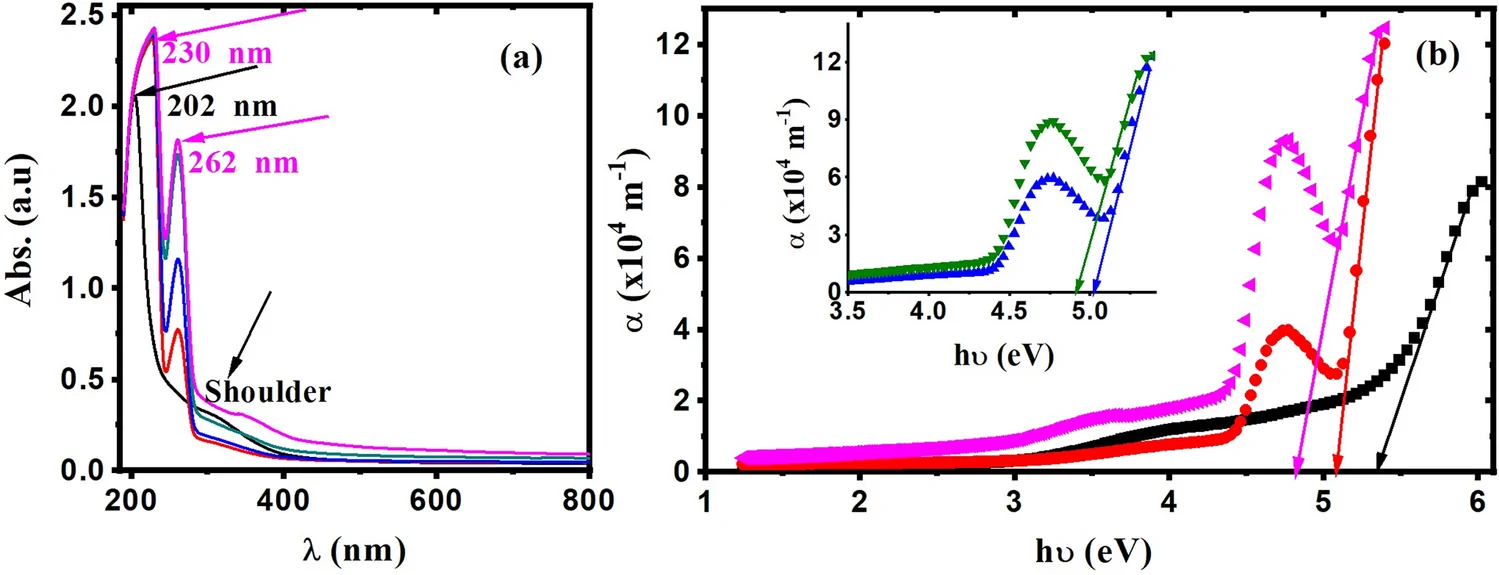
(**a**) absorption versus λ, (**b**) α versus hυ. ) pure PVA/Cs ) PVA/Cs/10wt% TEA-TOS, ) PVA/Cs/20wt% TEA-TOS, ) PVA/Cs/30wt% TEA-TOS, ) PVA/Cs/40wt% TEA-TOS.

The effect of TOS on the optical properties of PVA/CS blend is explored based on the UV/Vis measurements. Figure 10a illustrates the absorption behavior of PVA/Cs blend and PVA/Cs/TOS composites. It is noted that the absorption behavior of the mixture is characterized by the presence of an absorption peak at 202 nm, in addition to the presence of a shoulder in the range 280–315 nm. The absorption peak and shoulder are attributed to π–π* and n–π* transitions due to the presence of carbonyl group (C=O)^2,65^. Upon doping with TOS, the absorption peak of the PVA/Cs blend shifts to a higher wavelength at 230 nm, and the shoulder also shifts to the range of 310–350 nm. A new absorption peak at 262 nm was also observed in the composite samples. These features may be attributed to the complexation between TOS and the PVA/Cs matrix.

The absorption coefficient, α = 2.303 *A*/*d*; where *A* and *d* are the absorbance and sample thickness, is plotted against the photon energy for PVA/Cs blend and PVA/Cs/TOS composites to estimate the energy of the absorption edge (*E*_*ed*_) for these samples, as shown in Fig. 10b. By extrapolating the linear portions of Fig. 10b, the values of *E*_*ed*_ are determined and summarized in Table 4. It is observed that the *E*_*ed*_ values ​​gradually decrease with increasing TOS content, from 5.41 eV for PVA/Cs blend to 4.75 eV for highly doped sample, indicating that the bandgap energy value of these samples will also decrease, as will be seen later.

**Table 4:** The optical parameters of PVA/Cs/[ TEA-TOS] composites.

Samples	Optical parameters						
	*E*_*ed*_ (eV)	*E*_*U*_ (eV)	*β*	*E*_*e-p*_	*E*_*gd*_ (eV)	*E*_*gi1*_ (eV)	*E*_*gi2*_ (eV)
PVA/Cs	5.41	1.23	0.020	31.91	5.62	5.01	–
PVA/Cs/10wt% TEA-TOS	5.03	1.41	0.018	36.47	5.18	4.89	4.23
PVA/Cs/20wt% TEA-TOS	4.97	1.46	0.017	37.75	5.12	4.81	4.21
PVA/Cs/30wt% TEA-TOS	4.88	1.64	0.015	42.48	5.07	4.73	4.17
PVA/Cs/40wt% TEA-TOS	4.75	2.22	0.011	57.17	5.00	4.68	4.11

**Fig. 11:**
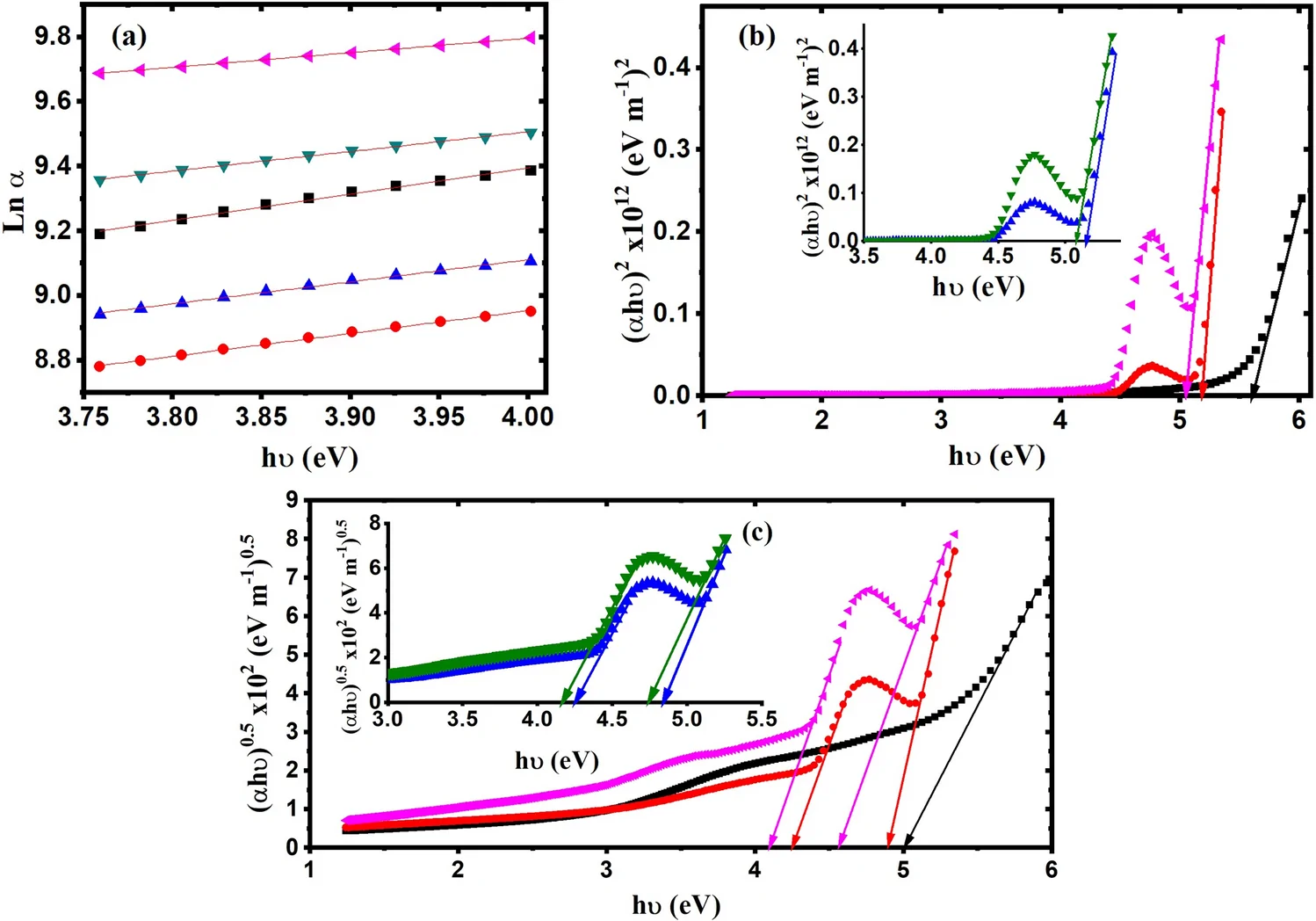
(**a**) Ln α versus hυ, (**b**) (αhυ)^2^ versus hυ and (**c**) (αhυ)^0.5^ versus hυ. ) pure PVA/Cs ) PVA/Cs/10wt% TEA-TOS, ) PVA/Cs/20wt% TEA-TOS, ) PVA/Cs/30wt% TEA-TOS, ) PVA/Cs/40wt% TEA-TOS.

The localized energy states and defects generated in PVA/Cs/TOS composites can be demonstrated by studying Urbach energy (*E*_*U*_), as follows^66^:

12 $$\alpha = \alpha_{0} \,\exp \left( {{\raise0.7ex\hbox{${h\upsilon }$} \!\mathord{\left/ {\vphantom {{h\upsilon } {E_{U} }}}\right.\kern-0pt} \!\lower0.7ex\hbox{${E_{U} }$}}} \right)$$

where α_0_ is a pre-exponential factor. Urbach energy displays an exponential dependence on the photon energy (hυ) and absorption coefficient. *E*_*U*_ values are estimated by plotting ln α against hυ and knowing the slope of the fitted lines, as shown in Fig. 11a. As listed in Table 4, the Urbach energy value increased from 1.23 eV for pure PVA/Cs blend to 2.22 eV for PVA/Cs/40wt% TOS composite. The increase in in *E*_*U*_ values indicates that the crystallinity of PVA/Cs blend is decreased upon increasing the TEA-TOS content, as mentioned in XRD section.

The steady increase in *E*_*U*_ values ​​with increasing TOS content indicates that the localized states and defects that act as trapping centers in the forbidden region of the PVA/Cs matrix have increased. Similar results are reported for many polymeric composites in the literature^67–69^. Therefore, PVA/Cs by doping with TOS salt and decreasing the absorption edge and hence the energy gap makes it a strong candidate for various optical and electronic applications.

The optical energy gap (*E*_*g*_) is a key factor in determining the distinctive properties of materials, as it plays a significant role in the design of new optoelectronic devices and solar cells. Therefore, its value is essential for accurately estimating what can be achieved using absorption spectra analysis of the optical samples studied. According to Mott and Davis, the optical band gap, whether direct (*E*_*dg*_) or indirect (*E*_*Ig*_), can be estimated by considering the dependence of the absorption coefficient on photon energy, as follows^70^:

13 $$\alpha h\upsilon = B(h\upsilon - E_{g} )^{x}$$

where *B* is a constant called the coefficient of the band-tail and *x* is an exponent equal to 1/2 or 2 depending on the electronic transition type, i.e., direct or indirect. Figure 11a, b demonstrates the dependence of (*αhυ*)^2^ and (*αhυ*)^0.5^ on the photon energy (*hυ*). By extrapolating the linear portions of the obtained curves to the x-axis, where α = 0, the values of (*E*_*dg*_) and (*E*_*Ig*_), ​​can be calculated. The obtained values ​​are given in Table 4. It is observed that, *E*_*dg*_ values decreased from 5.62 eV for pure PVA/Cs blend to 5.00 eV for PVA/Cs/40wt% TOS composite. On the other side, it is found that the PVA/Cs/TOS composites are dual-indirect bandgap. In the first region (I), the *E*_*ig*_ is decreased from 5.01 eV for pure PVA/Cs blend to 4.68 eV and to 4.11 eV in the second region for PVA/Cs/40wt% TOS composite, respectively. The dual band gap has been reported previously in polymeric nanocomposites. Fahmy et al. reported that chitosan/Fe^3+^ nanocomposite has dual band gap and the values of *E*_*g*_ decreased from 5.31 to 3.92 eV in the first region and to 3.19 eV in the second region^71^. Also, Abdelfattah et al. reported that PVA/Cs/ZnO nanocomposites have dual band gap and the value of *E*_*g*_ is decreased from 4.43 to 3.55 eV in the first region and to 3.15 eV in the second region^11^. It is reported that *E*_*g*_ PVA/Cs reduced from 5.39 to 4.45 eV after doping with 5wt% MWCNTs, also from 5.22 eV to 4.78 eV when doping with 1wt% AgNPs and from 5.954 to 5.783 eV after doping with 12wt% nanocurcumin^2,16,17^. Panda et al. published that the *E*_*g*_ of PVA-silver-ionic liquid (PVA-Ag-IL) composites is reduced from 3.38 eV for PVA to 2.06 eV for PVA-Ag-IL composite^25^. The decrease in the direct and indirect energy gap values ​​with increasing doping level is attributed to the strong complexation between TOS particles and PVA/Cs chains via O–H bonding, which produces localized states in the region between the valence band (LOMO) and the conduction band (HOMO) making it easier for electrons to transition between the two bands through these new energy levels.

**Fig. 12:**
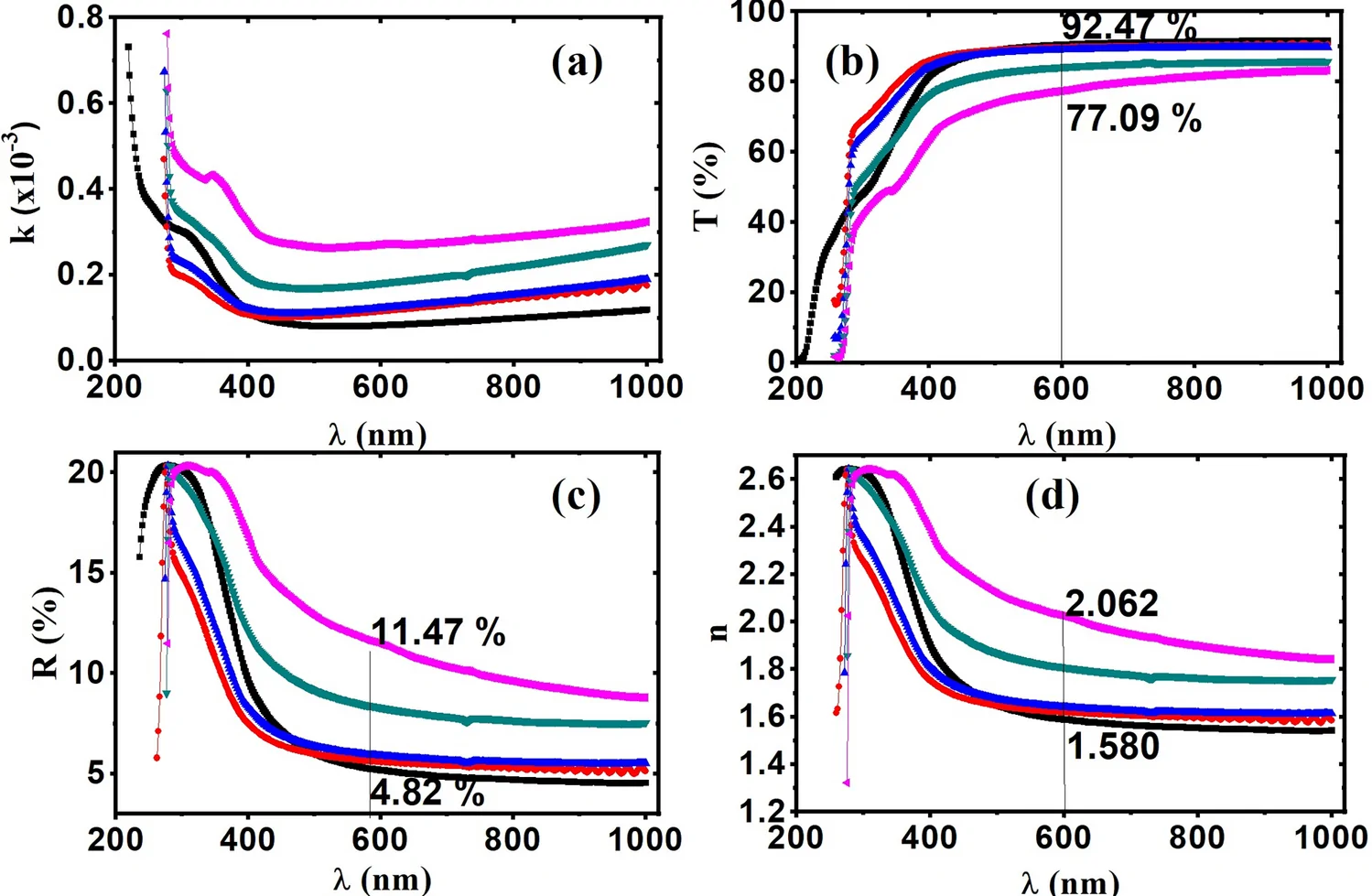
(**a**)* k* versus λ, (**b**) (T%) versus λ, (**c**) (R%) versus λ and (**d**) n versus λ. ) pure PVA/Cs ) PVA/Cs/10wt% TEA-TOS, ) PVA/Cs/20wt% TEA-TOS, ) PVA/Cs/30wt% TEA-TOS, ) PVA/Cs/40wt% TEA-TOS.

The extinction coefficient (*k* = *αλ/4π*) of PVA/Cs/TOS composites is plotted versus *λ*, as shown in Fig. 12a. It is observed that adding TOS at increasing ratios increased the *k* values due to the increase in charge carriers concentration and localized states resulting from TOS loading. It is evident that *k* decreases with increasing wavelength in the UV region, while it increases again with increasing wavelength starting at 430 nm in the visible light region. Furthermore, *k* values ​​increase linearly with λ in the high wavelength range. This indicates the importance of these composites for sensing applications in the visible region.

When a material is exposed to electromagnetic radiation, part of the light is absorbed, part is transmitted and the rest is reflected. Figure 12b, c illustrates the transmittance (T%) and reflectance (R%) versus *λ* for pure PVA/Cs blend and PVA/Cs/TOS composites. It is clear that with increasing TOS content, transmittance increases in the UV region while reflectance decreases, while in the visible light region both transmittance and reflectance tend to be constant with increasing wavelength. Interestingly, as the TOS content increases, transmittance increases and reflectance decreases. For example, at *λ* = 600 nm, the value of (T%) is reduced from 92.74 for pure PVA/Cs to 77.09 for PVA/Cs/40wt% TOS composite, whereas, (R%) is increased from 4.82 to 11.47.

The optical performance of a material is primarily determined by its refractive index (*n*) investigation to determine various optical features and their applications in specific optoelectronic devices. The refractive index can be expressed in terms of the values of *R* and *k* as follows^72^:

14 $$n = \frac{1 + R}{{1 - R}} + \left( {\frac{{\left( {1 + R} \right)^{2} }}{{\left( {1 - R} \right)^{2} }} - \left( {1 - k} \right)^{2} } \right)^{0.5}$$

According to Fig. 12d, it is observed that the *n* follows the absorbance behavior (Fig. 11a). In other words, the *n* in the UV region decreases sharply with increasing λ, while remaining almost constant in the visible region. Furthermore, it is observed that the *n* value of the pure PVA/Cs blend is increased as a result of TOS doping, enabling its use in advanced applications in optoelectronic devices. For example, the n value increases from 1.580 for the pure PVA/CS blend to 2.062 for PVA/Cs/40wt% TEA-TOS composite at λ = 600 nm. This enhancement in the values of *n* is attributed to the increased composites density and intermolecular bonds resulting from TOS doping. Similar behaviors are reported previously for many polymeric composites^73^.

### Single oscillator model (SOM)

**Fig. 13:**
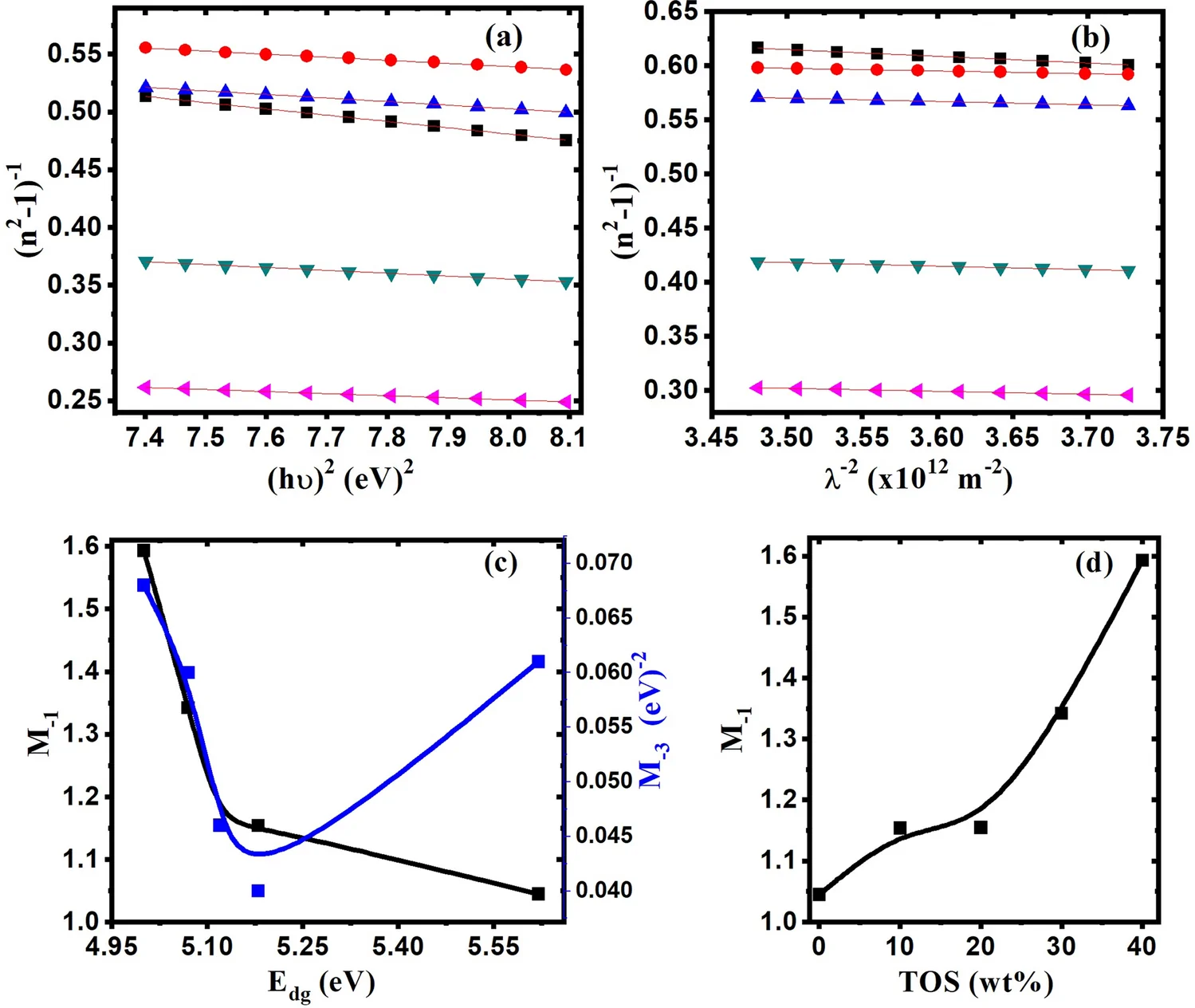
(**a**) (n^2^-1)^−1^ against (hυ)^2^, (**b**) (n^2^-1)^−1^ versus λ^−2^, (**c**) *M*_*−1*_ and *M*_*−3*_ versus E_dg_ and (**d**) *M*_*−1*_ versus TOS. ) pure PVA/Cs, ) PVA/Cs/10wt% TEA-TOS, ) PVA/Cs/20wt% TEA-TOS, ) PVA/Cs/30wt% TEA-TOS, ) PVA/Cs/40wt% TEA-TOS.

The refractive index dispersion of PVA/Cs/TEA-TOS composites below the optical absorption edge can be expressed in terms of the incident photon energy based on the SOM as follows^72^:

15 $$(n^{2} - 1)^{ - 1} = \frac{{E_{0} }}{{E_{d} }} - \frac{1}{{E_{d} E_{0} }}(h\upsilon )^{2}$$

where *E*_*o*_ represents the effective single oscillator energy, which in turn provides quantitative information about the overall band structure of the materials and *E*_*d*_ is the effective dispersion energy, which in turn also measures the average strength of interband optical transitions related to the structural order degree of the materials. Figure 13a depicts the variation of (n^2^-1)^−1^ versus (*hυ*)^2^ for the investigated samples. The intercept (*E*_*o*_*/E*_*d*_) and the slope (−*E*_*d*_*E*_*o*_)^−1^ of the fitted lines of Fig. 12a are used to estimate the values of *E*_*0*_ and E_d_ and listed in Table 5.

**Table 5:** Dispersion parameters of PVA/Cs/[TEA][TOS] composites.

Parameter	PVA/Cs	PVA/Cs/10wt% [TEA-TOS]	PVA/Cs/20wt% [TEA-TOS]	PVA/Cs/30wt% [TEA-TOS]	PVA/Cs/40wt% [TEA-TOS]
*E*_*0*_ (eV)	4.11	5.37	4.99	4.71	4.81
*E*_*d*_ (eV)	4.49	7.16	6.67	8.48	12.21
*n*_*0*_	1.44	1.52	1.53	1.67	1.88
*ε*_s_	2.09	2.33	2.34	2.80	3.53
*f* (eV)^2^	18.51	38.46	33.33	40.0	58.82
*M*_*−1*_	1.045	1.154	1.155	1.342	1.593
*M*_*−3*_ (eV)^−2^	0.061	0.040	0.046	0.060	0.068
*s*_*0*_ (x10^13^m^−2^)	1.612	4.166	3.333	3.125	3.703
*λ*_*0*_ (× 10^−7^m)	2.729	1.873	2.103	2.452	2.601
*ε*_L_	2.80	2.75	2.86	3.67	4.85
N/m*(× 10^56^ m^−3^ kg^−1^)	9.32	4.41	5.76	13.37	25.52
*N* (x10^27^m^−3^)	3.74	1.77	2.31	5.6	10.20
*ω*_p_ (× 10^15^ Hz)	1.64	1.13	1.29	1.96	2.71
*Ψ* (eV)	1.08	0.74	0.85	1.30	1.79
*E*_*P*_ (eV)	0.81	0.56	0.62	0.79	0.91
*E*_*f*_ (eV)	0.33	0.20	0.24	0.42	0.64
τ (× 10^–12^ s)	5.01	1.33	1.92	4.57	39.70
μ_opt_ (m^2^/s V)	2.00	0.531	0.767	1.82	1.59
ρ_opt_ (× 10^–8^ Ω m)	0.835	6.650	3.530	0.639	0.038

The interaction strength (*f*) between electromagnetic radiation and the material, static refractive index (*n*_*0*_) and static dielectric constant (*ε*_*s*_) PVA/Cs/TEA-TOS composites are calculated using the following equations and given in Table 5^72,73^.

16a $$n_{0} = \left( {1 + \frac{{E_{d} }}{{E_{0} }}} \right)^{1/2} ,$$

16b $$\varepsilon_{s} = n_{0}^{2} ,$$

16c $$f = E_{0} E_{d}$$

It is noted that these optical parameters have improved and their values ​​have increased with the increase in the content of TOS, which confirms that TOS has modified the electronic structure of PVA/Cs mixture. The optical transition moments (*M*_*−1*_ and *M*_*−3*_) are estimated for investigating the light-material interaction process using the following equation^74^:

17a $$M_{ - 1} = \frac{{E_{d} }}{{E_{0} }}$$

and

17b $$M_{ - 3} = \frac{{E_{d} }}{{E_{0}^{3} }}$$

The estimated values of (*M*_*−1*_ and *M*_*−3*_) are given in Table 5. Since both *M*_*−1*_ and *M*_*−3*_ depend mainly on *E*_*d*_ and *E*_*0*_, as well as *E*_*d*_ >  > *E*_*0*_, the values ​​of *M*_*−1*_ and *M*_*−3*_ should behave similarly to *E*_*d*_ with increasing TOS content. This behavior is attributed to the fact that the oscillator energy is directly proportional to the optical transition frequency^75^. Since *E*_*d*_ is attributed to the charge distribution in each unit cell as well as to the chemical bond which ensures that the optical properties depend on the material structure. Therefore, the high values of dispersion energy and transition moment indicate improved optical response structure of the material. It is also found that the values ​​of *M*_*−1*_ and *M*_*−3*_ decrease with increasing band gap energy as shown in Fig. 13c, while they increase with increasing TOS content, as shown in Fig. 13d.

To estimate the oscillator strength (*S*_*o*_) and the corresponding wavelength (*λ*_*o*_), the following Eq. (18) is obtained from Eqs. (15) and (16a)^75^:

18 $$\left( {n^{2} - 1} \right)^{ - 1} = \,\frac{1}{{s_{0} \lambda_{0}^{2} }} - \,\,\frac{1}{{s_{0} }}\,\lambda^{ - 2}$$

The values of *S*_*o*_ and *λ*_*o*_ are calculated from the intercept ($1/s_{o} \lambda_{0}^{2}$) and the slope (− 1/*s*_*0*_) of the fitted lines in Fig. 12b and listed in Table 5.

**Fig. 14:**
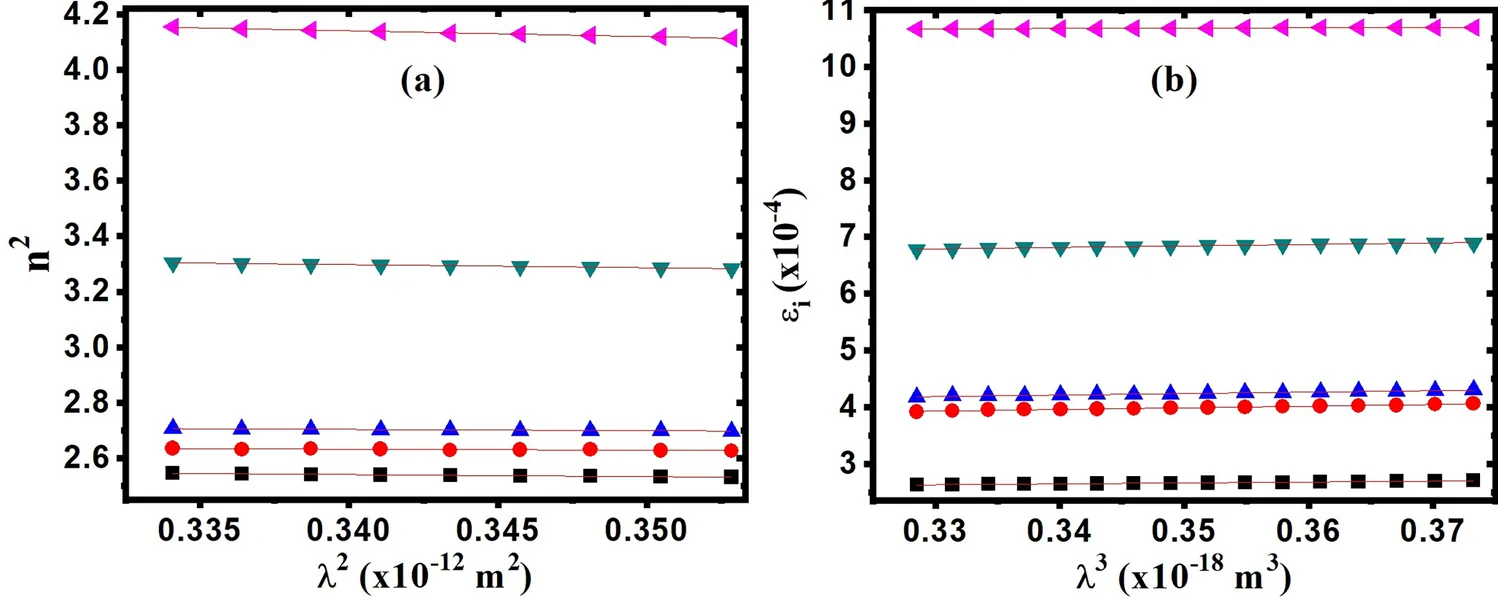
(**a**) n^2^ versus λ^2^ and (**b**) ε_i_ versus λ^3^. ) pure PVA/Cs, ) PVA/Cs/10wt% TEA-TOS, ) PVA/Cs/20wt% TEA-TOS, ) PVA/Cs/30wt% TEA-TOS, ) PVA/Cs/40wt% TEA-TOS.

According to Drude model, the correlation between real and imaginary parts (*ε*_*r*_ and *ε*_*i*_) of the optical dielectric constant and wavelength of the incident photon can be expressed as follows^76^:

19a $$\varepsilon_{r} = n^{2} = \varepsilon_{L} - \frac{{e^{2} }}{{4\pi \varepsilon_{0} c^{2} }}\left( {\frac{N}{{m^{*} }}} \right)\lambda^{2}$$

19b $$\varepsilon_{i} = \frac{1}{{4\pi^{3} \varepsilon_{0} }}\frac{{e^{2} }}{{c^{3} }}\left( {\frac{N}{{m^{*} }}} \right)\left( {\frac{1}{\tau }} \right)\lambda^{3}$$

where *ε*_*L*_ is the lattice dielectric constant, *e* is the electronic charge,* c* is speed of light, *N* is the charge carrier concentration, m^*^ is the electron effective mass (m^*^ = 0.44 *m*_*e*_) and *τ* is the relaxation time, respectively. Figure 14a illustrates the dependence of *n*^*2*^ on *λ*^*2*^ for all samples and (*ε*_*L*_) and (N/m^*^) values are estimated using the intercept and slope of the fitted lines of Fig. 14a, and summarized in Table 5. It is found that the higher the TOS content, the higher the *ε*_*L*_ ​​as well as the charge carrier concentration values. Also, the plasma frequency (*ω*_*p*_) are evaluated for PVA/Cs/TOS composites and listed in Table 5 by the following equation:

20 $$\omega_{p} = \sqrt {\frac{{e^{2} }}{{\varepsilon_{0} }}\left( {\frac{N}{{m^{*} }}} \right)}$$

Regarding the superposition between the classical free-electron model and the single oscillator model, some characteristic electronic parameters of the samples can be deduced, including the valence electron plasma energy (*Ψ*), the Penn energy (i.e., the average energy gap) (*E*_*P*_) and the Fermi energy (*E*_*f*_), as follow^77^:

21a $$\psi = \hbar \omega_{p}$$

21b $$E_{P} = {\raise0.7ex\hbox{$\psi $} \!\mathord{\left/ {\vphantom {\psi {\sqrt {(\varepsilon {}_{L} - 1)} }}}\right.\kern-0pt} \!\lower0.7ex\hbox{${\sqrt {(\varepsilon {}_{L} - 1)} }$}}$$

21c $$E_{f} = 0.2948 \times \psi^{{{\raise0.7ex\hbox{$4$} \!\mathord{\left/ {\vphantom {4 3}}\right.\kern-0pt} \!\lower0.7ex\hbox{$3$}}}}$$

The estimated values ​​of (*Ψ*, *E*_*P*_, and *E*_*f*_) summarized in Table 5, vary significantly with increasing [TEA][TOS] concentration. This variation demonstrates that the incorporation of [TEA][TOS] enhances the electronic structure of the PVA/Cs matrix, thereby affecting the optical and electrical properties of our investigated materials.

Figure 14b depicts the variation of ε_i_ versus *λ*^3^. The values of relaxation time (*τ*) are estimated using the slope of the fitted lines in Fig. 14b based on Eq. 19b and tabulated in Table 5. Moreover, the optical mobility (*μ*_*opt*_) and optical resistivity (*ρ*_*opt*_) of the PVA/Cs/TOS composites are estimated using the following equations and listed in Table 5^78^:

22 $$\mu_{opt} = \frac{e\tau }{{m^{*} }}\;\;{\mathrm{and}}\;\;\rho_{opt} = \frac{1}{{eN\mu_{opt} }}$$

### Linear/nonlinear optical properties

Among the most interesting and exciting properties of polymer composites is their nonlinearity, which expands their potential use in nonlinear devices. Nonlinear optical processes (NLOs) have numerous applications across various industries, including semiconductors and cell biology. Nonlinear materials are used in consumer goods such as medical lasers, optical sensors, DVDs, Blu-ray discs, and many others. When a material is exposed to electromagnetic radiation, the resulting polarization density (*P*) within the material can be described as follows^79^:

23 $$P = \chi^{(1)} E + \chi^{(2)} E^{2} + \chi^{(3)} E$$

Here, *E* is denoted by the electric field, the linear optical susceptibility by *χ*^(1)^, the second-order nonlinear optical susceptibility by *χ*^(2)^ and the third-order nonlinear optical susceptibility by *χ*^(3)^. The values of *χ*^(1)^ and *χ*^(3)^ are estimated using Miller’s empirical formula, as follows^80^:

24 $$\chi^{(1)} = (n_{0}^{2} - 1)/4\pi$$

and,

25 $$\chi^{(3)} = 1.79x10^{ - 10} \times (\chi^{(1)} )^{4} = \frac{{1.79x10^{ - 10} }}{{\left( {4\pi } \right)^{4} }}\left( {n_{0}^{2} - 1} \right)^{4}$$

The nonlinear refractive index (*n*_*2*_) also can be evaluated using *χ*^*(3)*^ as follows:

26 $$n{}_{2} = \frac{{12\pi \chi^{(3)} }}{{n_{0} }}$$

The values of *χ*^(1)^, *χ*^(3)^ and n_2_ are estimated and given in Table 6.

**Table 6:** Linear and nonlinear parameters of PVA/Cs/[TEA-TOS] composites.

Parameter	PVA/Cs	PVA/Cs/10wt% [TEA-TOS]	PVA/Cs/20wt% [TEA-TOS]	PVA/Cs/30wt% [TEA-TOS]	PVA/Cs/40wt% [TEA-TOS]
*χ*^(1)^	0.087	0.106	0.106	0.143	0.202
*χ*^(3)^ (× 10^–13^ esu)	0.104	0.227	0.229	0.758	2.984
n_2_ (× 10^–12^ esu)	0.267	0.560	0.564	1.706	5.979

**Fig. 15:**
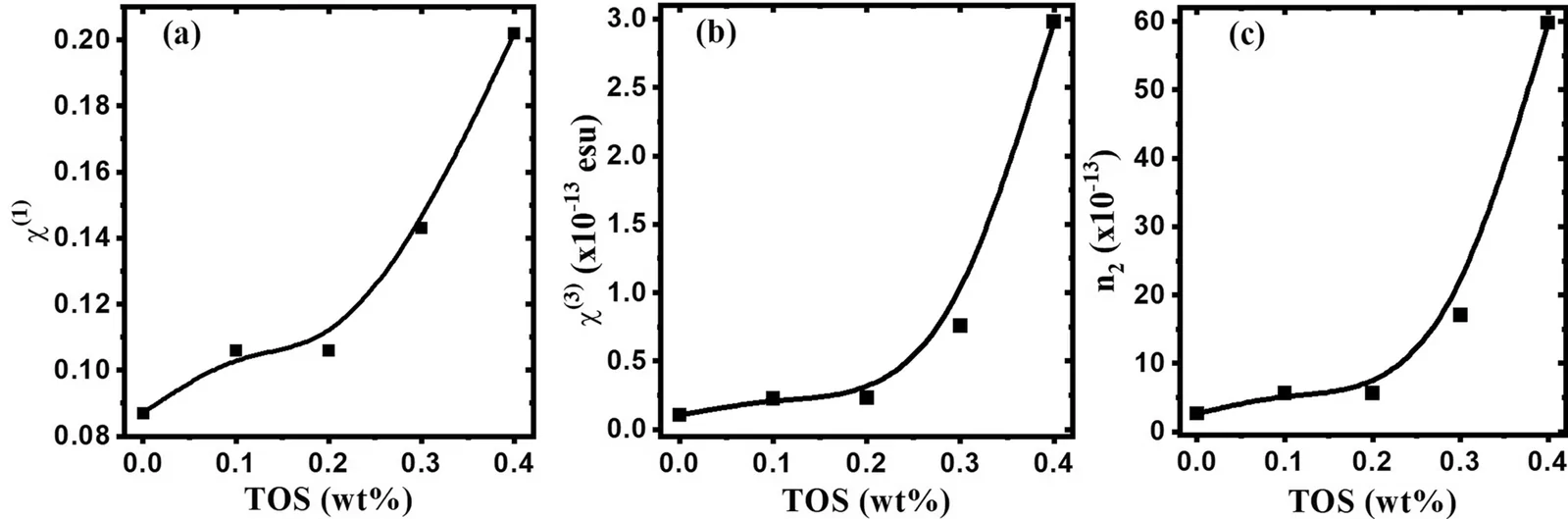
(**a**) χ^(1)^, (**b**) χ^(3)^ and (**c**) n_2_ versus TOS content.

Figure 15a–c depicts dependence of linear and nonlinear optical susceptibility and nonlinear refractive index on the TOS content. It is found that all these parameters are increased nonlinearly with increasing the TOS content. Similar results are reported for different materials^18,73^. It is published that *χ*^(3)^ values of PVVH/[TBA][BF_4_] ranged from 1.50 × 10^–15^ to 3.34 × 10^–15^ esu, from 4.93 × 10^–14^ to 9.54 × 10^–14^ esu for PVA/Cs/ZnO nanocomposites, from 0.80 × 10^–12^ to 2.22 × 10^–12^ esu for PVP/CMC/SnO_2_ZnS nanocomposites and from 0.025 × 10^–12^ to 4.874 × 10^–12^ esu for PVA/Cs/Ag nanocomposites^2,11,81,82^. The significant increases in these parameters can be attributed to the modification in the electronic band structure of the PVA/Cs host matrix resulting from the addition of TOS. The high NLO values ​​of the PVA/Cs blend upon the addition of TOS indicate the potential use of this composite in photonic and optoelectronic devices.

### Optical dielectric properties

The optical dielectric measurements are interpreted in terms of the complex permittivity i.e. real and imaginary permittivity as follows^83^:

27 $$\varepsilon^{*} = \varepsilon_{r} + j\varepsilon_{i}$$

where *ε*_*r*_ and *ε*_*i*_ are the real and imaginary components of the complex dielectric constant and are represented as follows^84^:

28 $$\varepsilon_{r} = n^{2} - k^{2} \;\;\;\;\varepsilon_{i} = 2nk$$

The dielectric constant of a material describes how it stores electrical energy. For polymer composites, this constant is determined by the interaction between the polymer matrix and the nanoparticles with a high dielectric constant, leading to increased polarization and thus a higher dielectric constant. Simultaneously, interfacial interactions and filler concentration significantly modify the dielectric permittivity and energy storage properties, improving performance in flexible electronics applications.

**Fig. 16:**
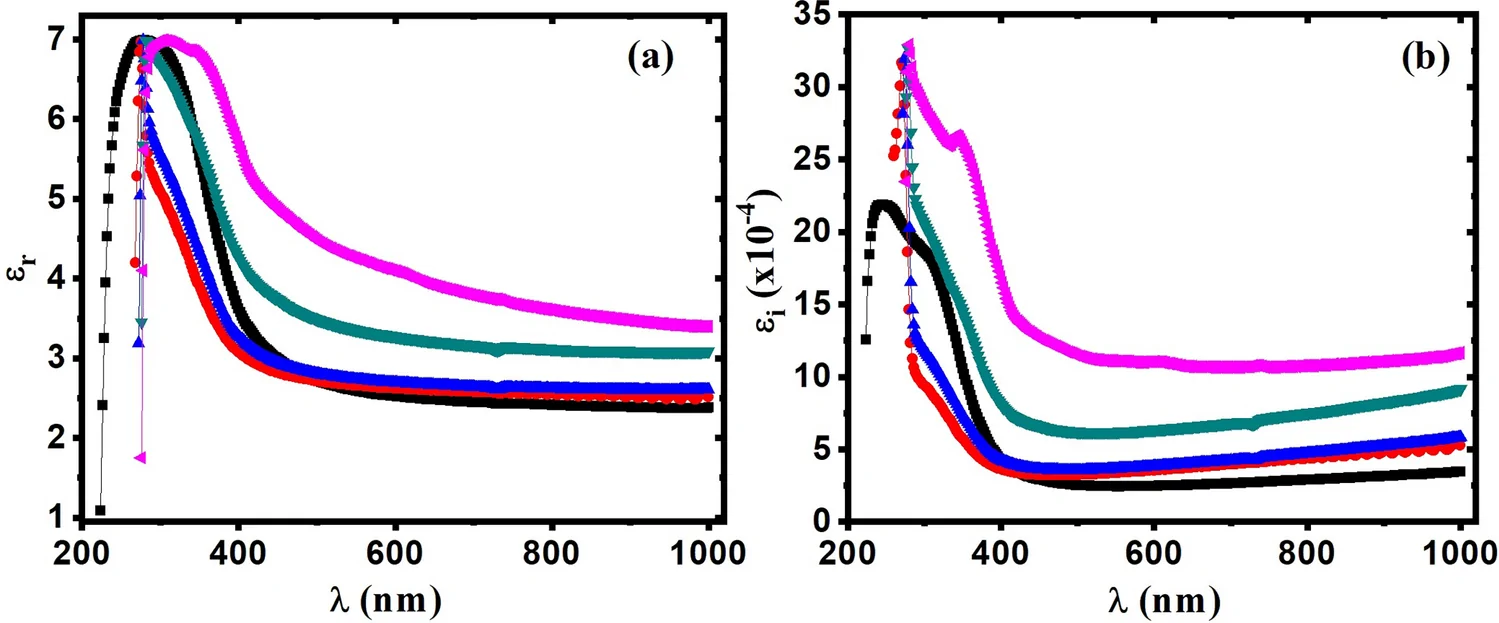
(**a**) ε_r_ versus λ, (**b**) ε_i_ versus λ, and (**c**) tan δ versus λ. ) pure PVA/Cs, ) PVA/Cs/10wt% TEA-TOS, ) PVA/Cs/20wt% TEA-TOS, ) PVA/Cs/30wt% TEA-TOS, ) PVA/Cs/40wt% TEA-TOS.

Figure 16a, b illustrates the behavior of *ε*_*r*_ and *ε*_*i*_ for all samples against the wavelength. It is observed that both *ε*_*r*_ and *ε*_*i*_ exhibited the same behavior. The higher values ​​of *ε*_*r*_ and *ε*_*i*_ in the low-wavelength region arise from increased contributions of charge carriers. Furthermore, the observed dispersion of *ε*_*r*_ and *ε*_*i*_ below 500 nm is related to the polar nature of the composite samples, which enables them to track changes in the incident electromagnetic field^85,86^. It is also observed that with the addition of varying amounts of [TEA-TOS], a significant increase in the dielectric constant is noted. This increase can be explained by an increase in the density of states, since *ε*_*r*_ is directly related to the density of states within the forbidden energy gap of the host polymer matrix. In other words, the increase in the dielectric constant indicates the addition of additional charge carriers to the host polymer matrix; consequently, the density of states increases.

### Optical electrical conductivity

The optical conductivity (σ_opt_) and electrical conductivity (σ_e_) are evaluated in terms of the values of absorption coefficient, refractive index and wavelength, as follows^87^:

29 $$\sigma_{opt} = \frac{\alpha nc}{{4\pi }}\quad \sigma_{e} = \frac{{2\lambda \sigma_{opt} }}{\alpha }$$

**Fig. 17:**
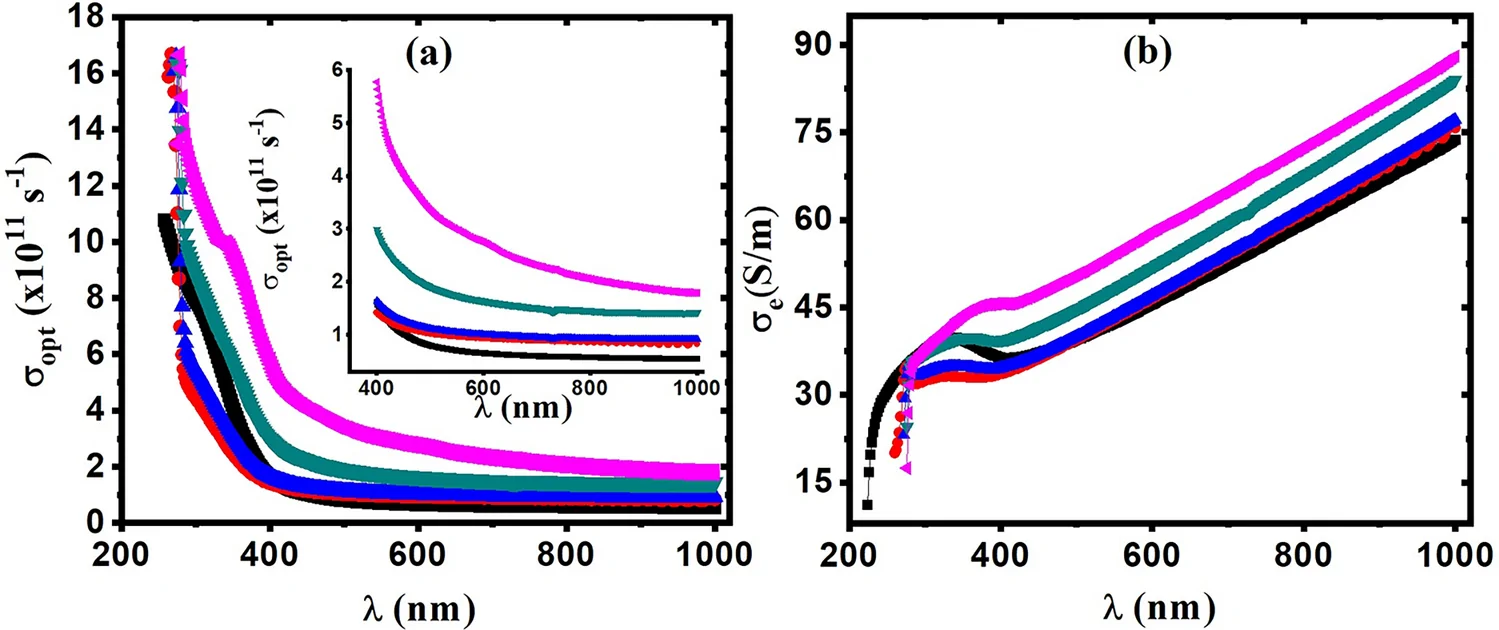
(**a**) σ_opt_ against λ and (**b**) σ_e_ against λ ) pure PVA/Cs, ) PVA/Cs/10wt% TEA-TOS, ) PVA/Cs/20wt% TEA-TOS, ) PVA/Cs/30wt% TEA-TOS, ) PVA/Cs/40wt% TEA-TOS.

Figure 17a shows the variation of the optical conductivity (σ_opt_) of pure PVA/Cs blend and PVA/Cs/TOS composites versus the wavelength of the incident photons. As can be seen the values of σ_opt_ are increased nonlinearly with increasing the content of TOS, as shown in the inset of Fig. 16a. This variation in σ_opt_ results from the new localized states generated in the forbidden gap, which made electrons move easily between those states. The decrease in the optical band gap also contributed to the increase in optical conductivity^88^. On other hand, the high values ​​of optical conductivity at lower wavelengths, i.e. higher energies, are attributed to the increased concentration of charge carriers resulting from the increased TOS content. Moreover, it is found that the electrical conductivity has increased from 11.33 to ~ 88.5 S/m with upon increasing the content of TOS, as shown in Fig. 17b, indicating the semiconducting nature of the PVA/Cs/TOS composites.

## Conclusion

XRD analysis exhibited that the crystallinity reduced from 55.65% for PVA/Cs blend to 29.82% for PVA/Cs/40 wt% TEA-TOS composite confirming the plasticizing and amorphizing effect of TEA-TOS. Deconvolution analysis of characteristic FTIR bands further revealed a reduction in crystallinity as the [TEA-TOS] content increased. TGA analysis showed that the thermal stability of PVA/Cs/TEA-TOS composites is enhanced with increasing the content of TEA-TOS. It is found that E_U_ increased from 1.23 to 2.22 eV, indicating decreased crystallinity in the composites, which supports the XRD findings. Enhanced light absorption, reduced bandgap energy, and an increased refractive index are observed and are attributed to the higher density of electronic states resulting from the incorporation of the TEA-TOS ionic liquid. SOM analysis also showed a significant enhancement in optical coefficients with increasing TEA-TOS content for example, *E*_*d*_, *ε*_*s*_ and *ε*_*L*_ are increased from 4.49 eV, 2.09, 2.80 to 12.21 eV, 3.53, 4.85, respectively. Also, all the linear/nonlinear optical parameters are improved with increasing the content of TEA-TOS. The significant improvement in the nonlinear optical parameters highlights the potential of PVA/Cs/TEA-TOS composites as promising materials for advanced photoelectronic applications.

## Data Availability

All data supporting the findings of this study are available within the paper. Datasets generated during the current are available from corresponding author reasonable request.
